# Automatic Differentiation for Explicitly Correlated MP2

**DOI:** 10.1021/acs.jctc.4c00818

**Published:** 2024-09-23

**Authors:** Erica C. Mitchell, Justin M. Turney, Henry F. Schaefer

**Affiliations:** † Department of Chemistry, University of Georgia, 302 East Campus Road, Athens, Georgia 30602, United States; ‡ Center for Computational Quantum Chemistry, University of Georgia, Athens, Georgia 30602, United States

## Abstract

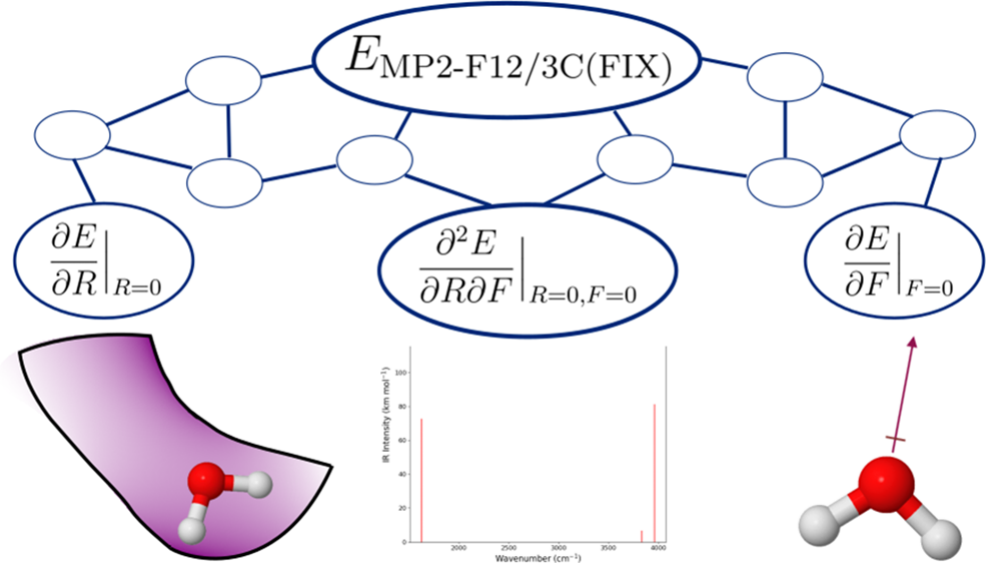

Automatic differentiation (AD) offers a route to achieve arbitrary-order derivatives of challenging wave function methods without the use of analytic gradients or response theory. Currently, AD has been predominantly used in methods where first- and/or second-order derivatives are available, but it has not been applied to methods lacking available derivatives. The most robust approximation of explicitly correlated MP2, MP2-F12/3C(FIX)+CABS, is one such method. By comparing the results of MP2-F12 computed with AD versus finite-differences, it is shown that (a) optimized geometries match to about 10^–3^ Å for bond lengths and a 10^–6^ degree for angles, and (b) dipole moments match to about 10^–6^ D. Hessians were observed to have poorer agreement with numerical results (10^–5^), which is attributed to deficiencies in AD implementations currently. However, it is notable that vibrational frequencies match within 10^–2^ cm^–1^. The use of AD also allowed the prediction of MP2-F12/3C(FIX)+CABS IR intensities for the first time.

## 1 Introduction

Explicitly correlated second-order Mo̷ller-Plesset perturbation theory (MP2-F12) has become a reliable tool for achieving highly accurate molecular ground-state energies^1−4^ and has been used to increase the accuracy of Monte Carlo simulations,^5,6^ double-hybrid density functionals,^7,8^ and symmetry-adapted perturbation theory.^9−12^ First developed in 1987 by Klopper and Kutzelnigg,^13,14^ MP2-F12 has seen tremendous advancements throughout its history.^15−19^ The development of the strong orthogonality projector, the standard approximations, and other ansätze have not only increased the rigor of the method but also its complexity as the combinations of approximations available result in over 216 possible variations.^20−22^ From numerous studies, the combination of Ansatz 3 [*Q̂*_12_ = (1 – *ô*_1_)(1 – *ô*_2_)(1 – *v̂*_1_*v̂*_2_)], Ansatz C (direct RI expansions), the fixed amplitude ansatz (FIX), and the complementary auxiliary basis set (CABS), which we denote as 3C(FIX)+CABS, has been determined to be the most robust among all the possible approximations.^21,23−28^

Although molecular ground-state energies have been the primary use for MP2-F12 methods, the inclusion of explicit correlation for analytic molecular properties has been investigated by various groups.^29−35^ Kordel, Villani, and Klopper were the first to apply gradient theory to MP2-F12 with the linear correlation factor () to obtain the relaxed one-electron reduced density matrix (1-RDM) and nuclear gradients.^29,30^ Soon after, Höfener, Hättig, and Klopper applied this same approach to modern density-fitted (DF) MP2-F12/3A*[T+V]+CABS showing results for both dipole moments, geometry optimizations, and the two-electron Darwin relativistic correction.^31−33^ In 2016, Zhang and co-workers attained the relaxed 1-RDM for MP2-F12/3C(FIX)+CABS but noted that the F12 contribution only agreed to at least 0.001 au with numerical computations due to the use of the 1-RDM to evaluate molecular properties.^34^ In 2017, Győrffy, Knizia, and Werner implemented DF-MP2-F12 analytic energy gradients with respect to nuclear coordinates and one-electron perturbations within the 3C(HY1, FIX) and 3C*(HY1, FIX) ansätze.^35^ In this context, Ansatz HY1 neglects all exchange contributions in one of the MP2-F12 equations, and the * indicates the use of the extended Brillouin condition.^22^ Although the CABS was not explicitly utilized in the equations, they used the union of the orbital and auxiliary basis sets to form their RI basis set. They then observed that the CABS singles correction was important to reaching high overall accuracy despite computing the correction via numerical differentiation. Due to the difficulties in getting the MP2-F12/3C(FIX)+CABS 1-RDM, to our knowledge, no attempts have been made to implement analytic gradients for the 3C(FIX)+CABS Ansatz with the CABS singles correction. In this work, we utilize automatic differentiation to compute the analytic derivatives for molecular properties, such as nuclear gradients and static multipole moments.

Automatic differentiation (AD) has been widely used in the computational sciences community for multiple years but has only recently been revived for quantum chemical applications.^36,37^ Quantum chemistry (QC) commonly uses derivatives to optimize molecular structures, determine molecular properties, and compute response properties. Currently, there are three main ways to compute a derivative in QC: (1) hand-derive the equations and hard-code them, (2) use finite-difference schemes, and (3) perform AD.^38^ Hand-derivation can be laborious, error-prone, time-consuming, and in some cases unfeasible. This can be shown by the fact that among the numerous correlated wave function methods in QC, only a handful have analytic derivatives available.^39^ Finite-difference methods have extended the ability to achieve derivatives of methods with no known analytic derivatives, but these can be computationally intractable and prone to numerical instabilities. AD differs from its predecessors by taking the advantage of the observation that any computer program can be broken down into a series of elementary operations (+, ∗, /, sin, etc.), where the derivative is known.^40,41^ This allows the exact derivative to be computed by composing the partial derivatives of each elementary operation in the program via the chain rule.

In the past years, AD has been applied to QC wave function methods in a variety of applications. The resurgence of AD for QC wave function methods can be attributed to the work of Tamayo-Mendoza, Aspuru-Guzik, and co-workers in 2018, where they developed a fully differentiable Hartree–Fock (HF) using the Python AD library AlgoPy^42^ to optimize basis sets.^37^ Soon after in 2020, Pavošević and Hammes-Schiffer used the Python AD library TensorFlow on the nuclear-electronic orbital coupled-cluster with doubles method to obtain derivatives with respect to the *t*- and λ-amplitudes.^43^ Later in 2021, Abbott, Schaefer, and co-workers developed Quax, which uses the Python AD library JAX^44^ to compute the high-order nuclear derivatives of standard QC methods such as, HF, MP2, and coupled-cluster with singles, doubles, and perturbative triples [CCSD(T)].^45^ In a different approach, Song, Martínez, and Neaton used AD in the QC package TeraChem to automatically generate diagrams for the analytic nuclear gradients for tensor-hypercontracted MP2 and complete active space second-order perturbation theory.^46^ In 2022, Kasim, Vinko, and Lehtola developed the software DQC, which stands for differentiable quantum chemistry.^47^ DQC uses the Python AD library PyTorch^48^ to allow differentiation with respect to any parameters present in the calculation, e.g., nuclear coordinates, atomic numbers, and electron occupation numbers. That same year, Zhang and Chan released PySCFAD, which relies on the AD library JAX.^44^ PySCFAD transforms the QC package PySCF to be AD compatible for electron-repulsion integrals (ERIs), HF, MP2, CC, full configuration interaction (FCI), and more.^49^

With the rising prominence of AD in computational chemistry, whether directly through studies like the above or indirectly through machine learning principles,^50^ questions still remain about the capability of AD technology to be used in conjunction with demanding and formidable quantum chemistry methods. Explicitly correlated methods present a promising test case for AD since for MP2-F12/3C(FIX), nuclear derivatives have remained elusive throughout the years and electric field derivatives differ from numerical results by 0.001 a.u. Herein, we present the nuclear and electric field derivatives for MP2-F12/3C(FIX) with the CABS by taking analytic derivatives of the energy using AD through the software Quax. With this, we aim to show that AD is a powerful tool that can enable the computation of vital molecular properties for highly sophisticated wave function methods.

## 2 Methods

### 2.1 MP2-F12/3C(FIX) Energy

In this section, we briefly review the basics of MP2-F12/3C(FIX)+CABS. MP2-F12 is a challenging method requiring multiple basis sets, various ansätze, and numerous tensor contractions. The 3C(FIX) Ansatz is the combination of three approximations: (1) the representation of the strong orthogonality projector, (2) the commutation between the kinetic and exchange operators with the correlation factor, and (3) the treatment of the geminal amplitudes. Detailed explanations of each of these approximations are given elsewhere,^15,22^ but the chosen approximations have been proven to make the 3C(FIX) Ansatz more robust than other ansätze.^18,23,27^ Of note, the SP-Ansatz or fixed amplitude ansatz of Ten-no used the coalesence conditions of the interelectronic cusp to set the amplitudes such that,^26,51^
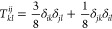
1The fixed amplitude ansatz is specified to be “diagonal orbital invariant”, which leads to a great reduction in computational complexity from  of Ansatz 3C to  of Ansatz 3C(FIX).^16,19^ In another ground-breaking advancement of F12 methods, the CABS approach was developed to improve the RI approximation by ensuring that the orbital basis set (OBS) is completely spanned by the auxiliary basis set (ABS).^28^ To form the CABS molecular orbital space, the procedure utilizes a singular value decomposition (SVD) to project the OBS out of the ABS (|*a′*⟩ = |*p′*⟩ – |*p*⟩). After applying the 3C(FIX) Ansatz and the CABS approach to the residuals from the minimization of the Hylleraas functional, the MP2-F12 intermediates can be determined as
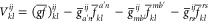
2
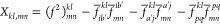
3
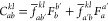
4
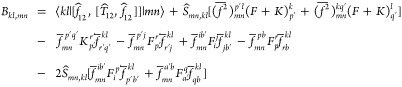
5where the indices are given in Table 1, and integral types are given in the Supporting Information, Table S1. Simplifying the residuals with the MP2-F12 intermediates and solving for the Hylleraas energy, the MP2-F12 energy can be shown to be an additive correction to the conventional MP2 energy,
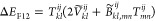
6where  and are
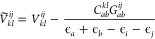
7
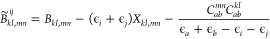
8

**Table 1:** Indices of the Matrix Elements in MP2-F12 Theory

type	description	acronym	indices
orbital basis set	all HF orbitals	OBS	*p*, *q*, *r*, *s*
	occupied HF orbitals		*i*, *j*, *k*, *l*, *m*, *n*
	virtual HF orbitals		*a*, *b*, *c*, *d*
auxiliary basis set	complementary auxiliary orbitals	CABS	*a′*, *b′*, *c′*, *d′*
RI basis set	all orbitals	RIBS	*p′*, *q′*, *r′*, *s′*
	all virtual orbitals		α, β, γ

Due to the introduction of the CABS, the basis set incompleteness error (BSIE) for the HF reference is non-negligible. To address the BSIE of HF, a perturbative approach is used, where the perturbation represents the relaxation of the HF orbitals in the presence of the CABS. Without this approach, the basis set error in the HF contribution becomes the primary source of error. In MP2-F12, this perturbation, known as the “CABS singles” correction, is entirely decoupled from the doubles energy correction, eq 6. By applying semicanonicalization of the CABS orbital space, the CABS singles correction can be determined to be
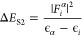
9where α runs over all virtual orbitals and the “S2” designates that the second-order singles correction is utilized.^52^

The total MP2-F12/3C(FIX)+CABS energy can then be broken down into three parts: (1) the conventional MP2 energy, *E*_MP2_, (2) the F12/3C(FIX)+CABS energy correction, Δ*E*_F12_, and the (3) CABS singles correction, Δ*E*_S2_,
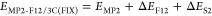
10

### 2.2 Automatic Differentiation

**Figure 1:**
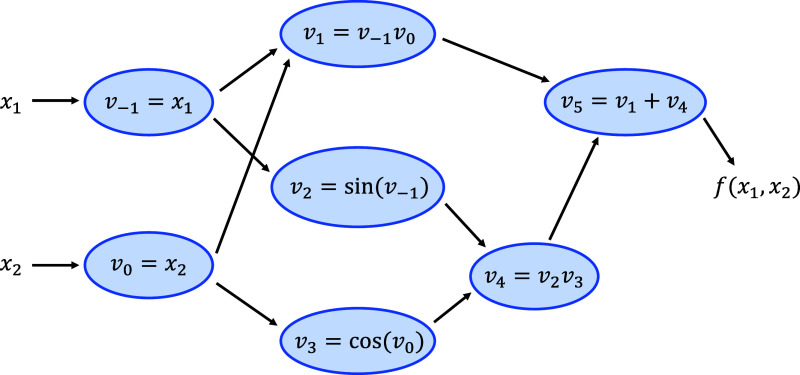
Computational graph of *f*(*x*_1_, *x*_2_) = *x*_1_*x*_2_ + sin(*x*_1_) cos(*x*_2_).

In considering derivatives for MP2-F12/3C(FIX)+CABS, automatic differentiation (AD) provides an elegant route to compute them. There are two modes in which AD may be done forward- and backward-mode, but this study focuses on the use of forward-mode. To understand forward-mode AD, take the equation,
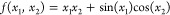
11and its computational graph, Figure 1. Each *v*_*i*_ is an intermediate in the computation that is subject to one elementary operation (+, ∗, /, sin, etc.) and, in forward-mode AD, every *v*_*i*_ has an associated derivative, e.g., . To compute each *v̇*_*i*_, an AD library is utilized to perform the derivative rules associated with the elementary operation.

Currently, Python AD libraries are the most popular with a wide-range of users, large development teams, and multiple functions available. PyTorch^48^ and TensorFlow^53^ are prominent libraries in machine learning with extensive AD capabilities, but JAX is a newer alternative developed by Google, which focuses more on the AD functionality.^44^ To be precise, JAX is a domain-specific tracing JIT (just-in-time) compiler that generates high-performance accelerator code from pure Python, NumPy, and SciPy functions.^54^ In more colloquial terms, JAX improves computational performance by optimizing the code compilation using Google’s accelerated linear algebra compiler (XLA), which enables Python functions to be evaluated efficiently. For the purposes of AD, JAX provides an easy user interface that transforms traditional NumPy and SciPy functions into composable functions that can be automatically differentiated in either forward- or reverse-mode.

In quantum chemistry, both PySCFAD and Quax were developed using JAX to obtain arbitrary-order derivatives of electronic structure methods. We chose to develop the MP2-F12/3C(FIX) derivatives in Quax due to its use of Libint2^55^ as the integral backend, since the necessary integrals are not available through Libcint.^56^ By utilizing a C++ library and JAX’s user-defined primitives, the computational time and memory requirements were able to be reduced significantly by employing: (a) integral symmetries, (b) integral screening, (c) OpenMP threading, and (d) disk algorithms. The increased computational prefactor and memory requirements of F12 methods due to the new integral types from the correlation factor is especially helped by the option of disk capability. The choice of Quax then provides the best environment to develop arbitrary-order derivatives for explicitly correlated MP2 over alternatives.

## 3 Results and Discussion

Molecular properties can be understood as the response to an applied perturbation,^39,57^
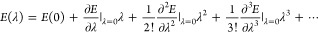
12Here, we take the analytic derivative of the energy with respect to geometric and electric field perturbations using automatic differentiation (AD). This means that only knowledge concerning how to implement the MP2-F12 energy within the 3C(FIX)+CABS Ansatz is needed. To compute the MP2-F12 energy, we have implemented the method into both Quax,^45^ for the AD, and Psi4,^58^ for numerical evaluation.

For MP2-F12, both programs used the cc-pVXZ-F12 and aug-cc-pVXZ basis sets with appropriate correlation parameters and CABS (cc-pVXZ-F12-OptRI and aug-cc-pVXZ-OptRI, respectively).^59−61^ Conventional MP2 used the standard aug-cc-pVXZ basis sets (X = D, T, Q) with tight *d* functions, aug-cc-pV(X + d)Z, on the second-row atoms where appropriate.^62−64^ Tight *d* functions were also included for MP2-F12 when the standard aug-cc-pVXZ basis was used. Although different basis sets are used between MP2-F12 and MP2, the MP2-F12/cc-pVXZ-F12 have been repeatedly shown to attain MP2/aug-cc-pV(X + 2)Z quality results for correlation energies.^1,2,65^ We abbreviate the cc-pVXZ-F12 and aug-cc-pVXZ basis sets as VXZ-F12 and aVXZ (X = D, T, Q, 5) from this point forward. To compare the properties of MP2-F12/VXZ-F12 and MP2-F12/aVXZ along with the MP2/aVXZ properties, the desired property was computed with the MP2 complete basis set (CBS) limit as a reference. The CBS extrapolations use Feller’s three-point extrapolation (X = T, Q, 5) for the SCF computations and Helgaker’s two-point extrapolation (X = Q, 5) for the correlated computations. All quantities were computed using the frozen-core approximation.

### 3.1 Geometry Optimizations

Geometry optimizations were done using the test set from Győrffy, Werner, and Knizia, which includes the following 16 molecules: H_2_O, H_2_S, CH_2_O, NH_3_, HNO, HOF, HCN, HNC, HCP, HBS, HF, HCl, CO, SiO, PN, and CS.^35^ One molecule has been removed from the set because Quax does not currently have the capability to determine excited-state geometries, CH_2_ (^1^*A*_1_). All equilibrium bond lengths, angles, and torsion angles are presented in the Supporting Information (SI), Tables S2–S17.

All optimized geometries were obtained using pyOptKing^66^ to read in the appropriate energies and gradients through Psi4’s finite difference driver.^58^ Each geometry was also optimized with rigorous convergence criteria: maximum element of the gradient (1.5 × 10^–5^), root-mean-square of the gradient (1.0 × 10^–5^), maximum element of displacement (6.0 × 10^–5^), and the root-mean-square of displacement (4.0 × 10^–5^). Quax was utilized to obtain the MP2-F12 nuclear gradient by AD while Psi4 was utilized to approximate the nuclear gradient with finite differences (FINDIF) of MP2-F12 energies according to a 5-point scheme with 0.001 a.u. displacements. Psi4 nuclear gradient computations for MP2 were computed using the same FINDIF of energies as the MP2-F12 gradients, since the frozen-core approximation was utilized for all levels of theory. For the MP2/CBS frozen-core geometry optimization, extrapolation was done using focal point analysis with a three-point extrapolation for the analytic SCF gradients and a two-point extrapolation for the 5-point MP2 finite difference gradient.

The AD geometries were first compared to the geometries obtained by finite differences, Table 2. From both mean absolute deviations (MADs) and mean signed deviations (MSDs), the AD and FINDIF geometries can be considered equivalent matching at about a micro-Angstrom for bond lengths and less than a milli-degree for angles. It is promising to observe that the linear angles are being correctly determined using the AD despite no symmetry being enforced. The excellent agreement between the AD and FINDIF optimized geometries suggests that AD is a suitable replacement for finite difference schemes to achieve optimized geometries.

**Table 2:** Mean Absolute Deviations (MADs) and Mean Signed Deviations (MSDs) of the 16-Molecule Test Set for the Bond Lengths, Bond Angles, Torsion Angles, and Linear Angles Comparing MP2-F12 Optimized Geometries from Automatic Differentiation to Those Obtained Using Finite Differences of Energies

parameter	VDZ-F12		aVDZ	
	MAD	MSD	MAD	MSD
bond lengths (Å)	2 × 10^–6^	5 × 10^–7^	1 × 10^–5^	8 × 10^–6^
bond angles (deg)	1 × 10^–4^	5 × 10^–5^	7 × 10^–5^	5 × 10^–5^
torsion angles (deg)	2 × 10^–4^	–6 × 10^–5^	1 × 10^–4^	–4 × 10^–5^
linear angles (deg)	0.00	0.00	0.00	0.00

With the confirmation of AD for the MP2-F12 nuclear gradients, the basis set convergence of the MP2-F12 optimized geometries was assessed for both the VDZ-F12 and aVDZ basis sets. Table 3 presents the MADs while Table 4 presents the MSDs. Both statistical comparisons show that MP2-F12/VDZ-F12 and MP2-F12/aVDZ can provide geometries near MP2/aVQZ quality or better. Between MP2-F12 with VDZ-F12 or aVDZ, the VDZ-F12 geometries appear to have a slightly better convergence toward the CBS limit, especially for torsion angles.

**Table 3:** Mean Absolute Deviations (MADs) of the 16-Molecule Test Set for the Bond Lengths, Bond Angles, Torsion Angles, and Linear Angles^a^

Parameter	MP2-F12	MP2-F12	MP2	MP2	MP2
	VDZ-F12	aVDZ	aVDZ	aVTZ	aVQZ
bond lengths (Å)	0.001	0.002	0.014	0.003	0.001
bond angles (deg)	0.06	0.07	0.49	0.14	0.09
torsion angles (deg)	0.05	0.12	1.06	0.20	0.12
linear angles (deg)	3 × 10^–5^	3 × 10^–5^	2 × 10^–5^	3 × 10^–5^	3 × 10^–5^

a Reference geometries are optimized at MP2/aV[TQ5, Q5]Z level of theory.

**Table 4:** Mean Signed Deviations (MSDs) of the 16-Molecule Test Set for the Bond Lengths, Bond Angles, Torsion Angles, and Linear Angles^a^

parameter	MP2-F12	MP2-F12	MP2	MP2	MP2
	VDZ-F12	aVDZ	aVDZ	aVTZ	aVQZ
bond lengths (Å)	–7 × 10^–4^	–0.002	–0.014	–0.003	–4 × 10^–4^
bond angles (deg)	0.03	0.04	0.46	0.11	0.05
torsion angles (deg)	–0.02	–0.04	–0.35	–0.07	–0.04
linear angles (deg)	6 × 10^–6^	6 × 10^–6^	9 × 10^–6^	2 × 10^–5^	6 × 10^–6^

a Reference geometries are optimized at MP2/aV[TQ5, Q5]Z level of theory.

### 3.2 Multipole Moments

To demonstrate the ability of Quax to compute multipole moments, the dipole moment (μ) of the same 16 molecule test set from the geometry optimizations was used. A majority of the test set only have a nonzero component for μ_*z*_ with the exception of HNO and HOF. For consistency, only the μ_*z*_ moment was considered for these two molecules as well, to confirm that AD could correctly predict the trivial μ_*z*_. The μ_*z*_ for all geometries are presented in the SI, Tables S18 and S19.

The geometry for the μ_*z*_ computations was taken from the NIST Computational Chemistry Comparison and Benchmark Database (CCCBDB).^67^ To assess the quality of the AD computations, the results from Quax were compared to dipole moments obtained through FINDIF of energies from Psi4 and analytic relaxed 1-RDMs from MPQC 3.0.0.^68,69^ Due to the significant discrepancy of the MPQC μ_*z*_ from numerical results in the study by Zhang et al.,^34^ the finite difference computations were taken as the reference. For all MP2 computations, the FINDIF were done using a 5-point scheme with a perturbation of 0.0005 a.u. The comparison of the AD and analytic results to the FINDIF is presented in Table 5. The mean absolute errors (MAEs) between the FINDIF and exact methods reveal that the use of AD should be considered over the analytic relaxed 1-RDM. Both AD and analytic results match the numerically obtained Hartree–Fock dipole moment to better than 10^–8^ a.u. The analytic derivatives achieve much closer accuracy to the finite-difference MP2 μ_*z*_, and this is suspected to be due to the limitations of AD as energies match within 8 or 9 digits. The analytic CABS correction is comparable to the accuracy of the AD, but the AD is slightly better. For the F12 correction, the AD is drastically closer to the numerical results with better than 10^–6^ a.u. agreement. Our observations for the analytic F12 contribution match those stated by Zhang and co-workers, where they report that “for the F12 correction, the agreement is only 0.001 a.u. or better.”^34^

**Table 5:** Mean Absolute Error (MAE) of the AD and Analytic μ_*z*_ for the 16-Molecule Test Set with Respect to Numerical Results^a^

basis set	algorithm	Δ_HF_	Δ_MP2_	Δ_S2_	Δ_F12_
VDZ-F12	AD	5 × 10^–9^	6 × 10^–6^	2 × 10^–7^	5 × 10^–7^
	analytic	8 × 10^–9^	8 × 10^–9^	1 × 10^–5^	6 × 10^–4^
aVDZ	AD	7 × 10^–9^	7 × 10^–6^	8 × 10^–7^	1 × 10^–6^
	analytic	1 × 10^–9^	6 × 10^–9^	4 × 10^–6^	9 × 10^–4^

a Units in a.u.

**Figure 2:**
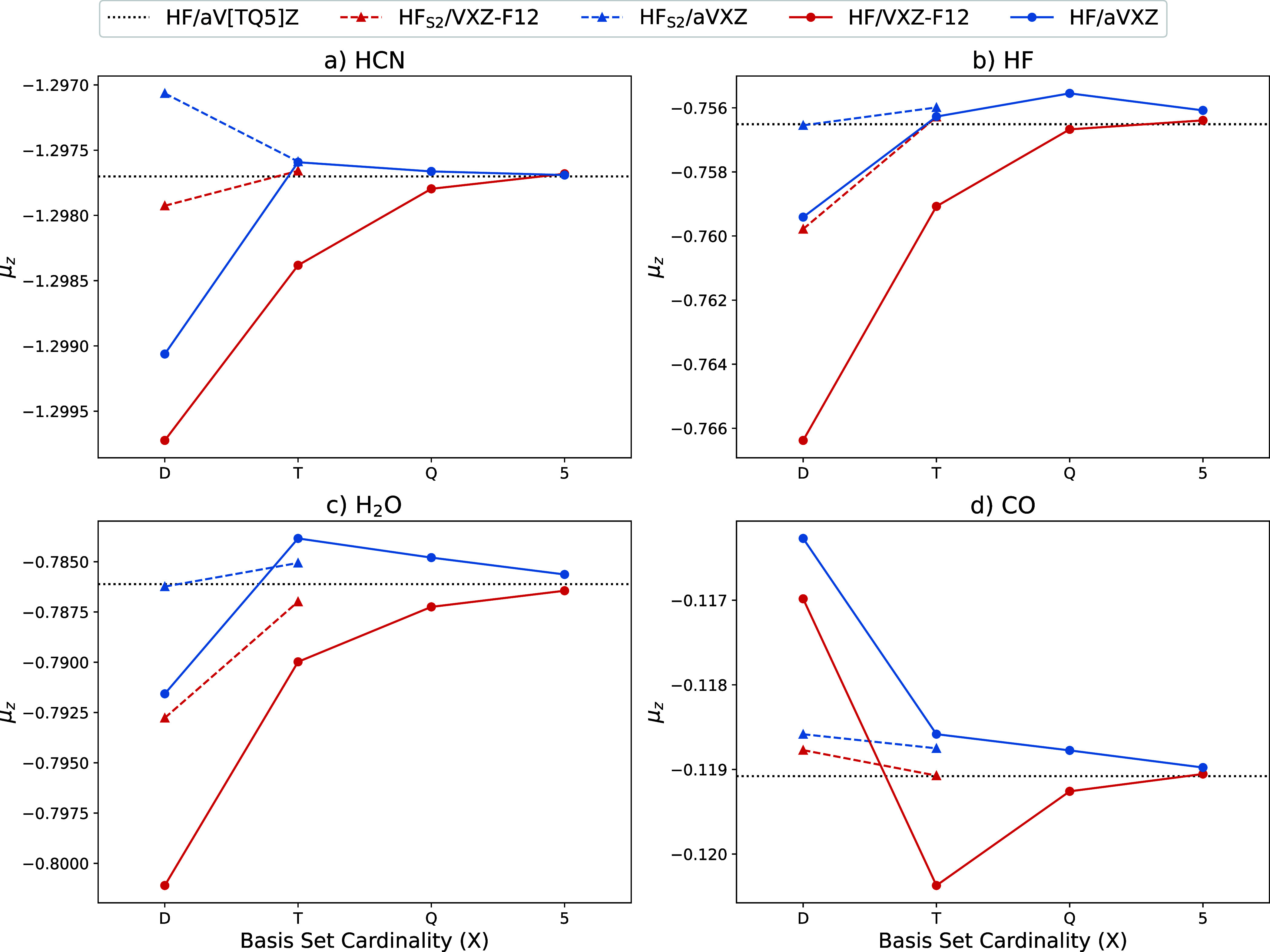
Basis set convergence of the HF and the CABS-singles corrected HF (HF_S2_) contributions (a.u.) to μ_*z*_ for a subset of the test molecules.

**Figure 3:**
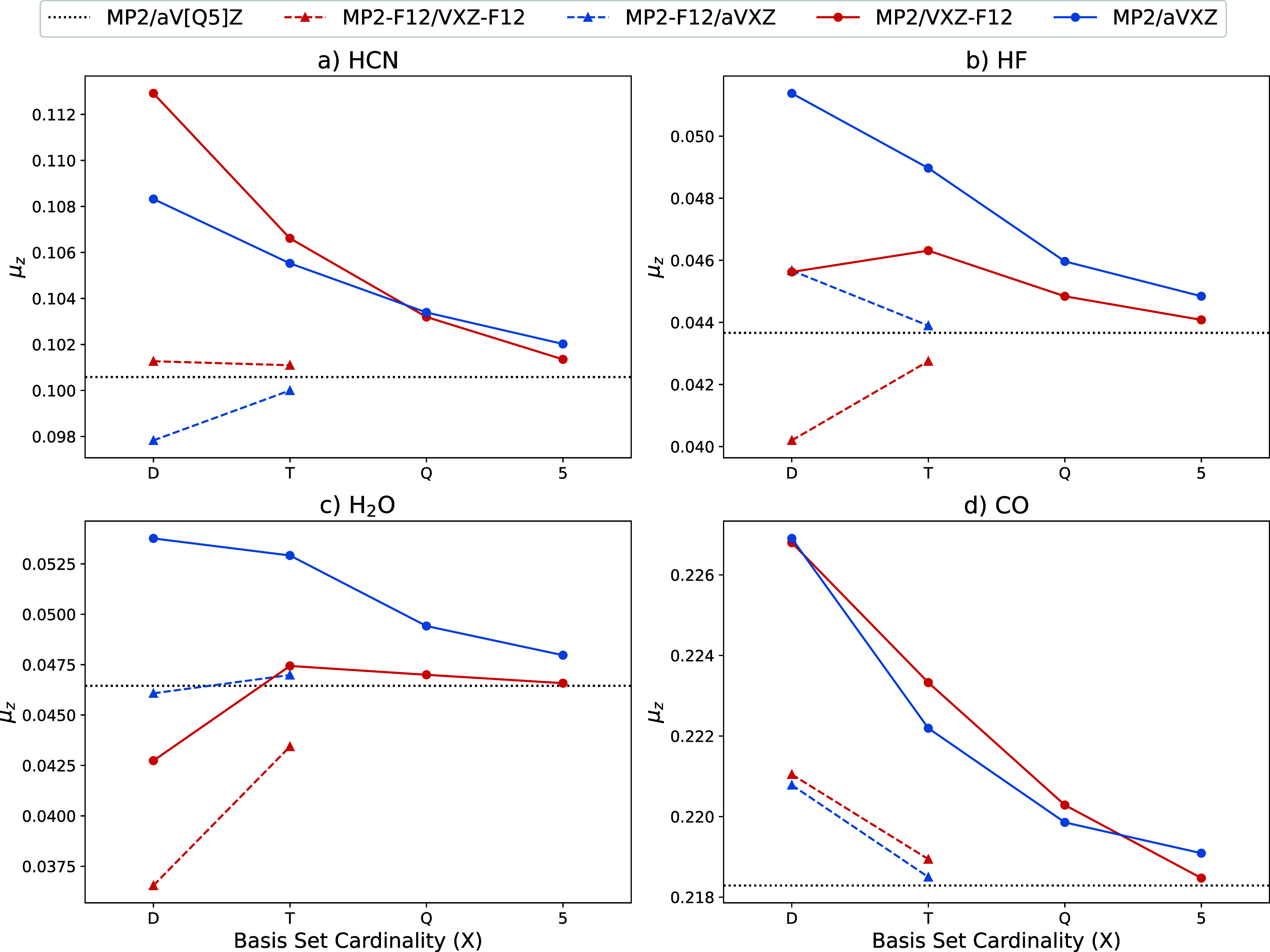
Basis set convergence of the MP2 and F12 contributions (a.u.) to μ_*z*_ for a subset of the test molecules. The F12 contribution does not include the CABS singles correction.

We further compare the AD obtained μ_*z*_ to the finite-difference results by observing the basis set convergence of the uncorrelated contributions (Figure 2) and correlated contributions (Figure 3) of the total μ_*z*_. This is presented for only a subset of the molecules (HCN, HF, H_2_O, and CO) to provide a comparison to the test set of Zhang et al.^34^ while the full set are given in the SI, Figures S1–S14. For the uncorrelated contributions, Figure 2, the CABS singles correction appears to be most important for the aVDZ and VDZ-F12 basis sets. This correction still has significance for the VTZ-F12 basis set but provides very little, if any, improvement for the aVTZ basis set. The basis set convergence of both basis set families is not truly observed until the triple ζ cardinality is utilized with the double ζ cardinality providing large errors, especially for VDZ-F12.

For the correlated contributions, Figure 3, the F12 correction increases the convergence by about X + 2 cardinality on average. This accelerated convergence mainly holds for the aVXZ basis set family with the VXZ-F12 family having a more variable F12 correction. In particular, for H_2_O, the F12 correction with VXZ-F12 seems to worsen the μ_*z*_ with respect to the MP2/CBS limit. Although the MP2/VXZ-F12 correlation contributions appear to converge faster to the CBS limit, the MP2-F12/aVXZ contributions perform much better than MP2-F12/VXZ-F12. The improved performance of aVXZ over VXZ-F12 was also observed by Zhang and co-workers.^34^ This result is in contrast to the accuracy of VXZ-F12 for absolute correlation energies and nuclear gradients, but is consistent with the conclusions for reaction barriers, reaction energies, and atomization energies.^70^

### 3.3 IR Intensities

With the assurance that both the nuclear and electric field derivatives achieved by AD are highly accurate, the mixed derivatives were considered as well. To best show this, we present the infrared (IR) intensities of a subset of the 16-molecule test set: H_2_O, H_2_S, HF, HCl, CO, SiO, PN, and CS. To obtain IR intensities, both the Hessian and Cartesian dipole derivatives are needed. The memory requirements for the Hessian limited our choice of molecules to mainly diatomics. Overall, the IR test set contains 12 frequencies/intensities, which are presented in the SI (Tables S21 and S22) along with the MP2-F12 IR spectra (Figures S15–S22).

The Hessian was first examined to evaluate the accuracy of the AD. Again, the AD was performed with Quax while Psi4 was utilized to compute the explicitly correlated Hessian with a 5-point FINDIF scheme with 0.001 a.u. displacements. To compare the AD and FINDIF Hessians, the RMSD was calculated and the average RMSD for the IR test set was 1.04 × 10^–5^ a.u. Despite the poorer agreement between the Hessians, the AD and numerical frequencies have a very low MAE of 0.04 cm^–1^.

To analyze the basis set convergence of the frequencies and IR intensities, ORCA 5.0.4^71−73^ was used to compute numerical quantities and Quax was used for AD. The numerical IR intensities were computed using FINDIF of MP2 energies to obtain the Hessian and analytic dipole derivatives. ORCA uses a 3-point central difference scheme with 0.005 au displacements for the Hessian. Table 6 presents the MAEs of the frequencies and intensities for MP2/aVXZ (X = D, T, Q) and MP2-F12/aVDZ compared to MP2/aV5Z. In contrast to nuclear gradients and dipole moments, the MP2-F12 frequencies and intensities exhibit slower convergence to the CBS limit, where the MP2-F12/aVDZ quantities tend to fall between the quality of MP2/aVTZ and MP2/aVQZ.

**Table 6:** Mean Absolute Errors (MAEs) for the Vibrational Frequencies and IR Intensities of the IR Test Set^a^

parameter	MP2-F12	MP2	MP2	MP2
	aVDZ	aVDZ	aVTZ	aVQZ
frequencies (cm^–1^)	9.12	52.69	12.69	2.72
IR intensities (km mol^–1^)	0.86	3.37	1.32	0.40

a Reference frequencies and intensities were taken from the MP2/aV5Z level of theory.

The shortcomings of the AD with respect to higher-order differentiation could be attributed to two factors: (1) degeneracies in the Hartree–Fock orbitals and (2) the use of the null space in the CABS procedure. Both of these reveal a major disadvantage of automatic differentiation. It is well-known that degenerate eigenvalues in an eigendecomposition present a challenge for AD algorithms.^37,45,49,74−76^ AlgoPy used univariate Taylor arithmetic to remove degeneracies, but the program is deprecated.^42^ JAX has an experimental module which allows higher-order automatic differentiation through propagation of truncated Taylor polynomials, but its current state does not have support for eigendecompositions.^77^ In particular for a SVD, the null space presents an issue for AD since the choice of vectors for the null space is arbitrary, and therefore its derivative is not well-defined. Although both the null space and repeated eigenvalues do not have well-defined derivatives, degeneracies are often lifted by inserting a small amount of noise to slightly shift the eigenspectrum, but this may not be sufficient if degeneracies arise in the derivative eigenvalues as well.^45,75,78^ Solutions to this problem are outside of the scope of this work, but advances in automatic differentiation seem to be promising in this area.^79−83^

Furthermore, with high-order differentiation, issues arise in terms of performance. For our implementation of forward-mode AD in Quax, all derivatives of the two-electron integrals need to be held in memory until no longer needed. While a detailed performance analysis was not the primary goal of this study, Abbott and co-workers meticulously examined the performance of Quax, noting that peak memory usage increased rapidly.^45^ For example, they report that CH_2_O at the MP2/cc-pVTZ level of theory required 3.0 GB for the energy calculation, 32.1 GB for the full gradient, and a hefty 372.1 GB for the full Hessian computation. When determining the peak memory usage for MP2-F12, these numbers escalate even faster due to the introduction of four new integral types and the CABS. Specifically, the computation and storage of two-electron integrals require significantly more resources for F12 methods, which contributes to the rapid rise in memory requirements. The largest computation in this study was the MP2-F12/aVDZ full Hessian for SiO, which peaked at roughly 371 GB on a single AMD EPYC node of the Sapelo2 cluster at the Georgia Advanced Computing Resource Center. Although JAX was used in the current study, the prospect of using lower-level AD libraries, such as Enzyme,^84−86^ to alleviate computational burden is of high interest for the future of AD for highly sophisticated QC methods.

## 4 Conclusions

In this work, the MP2-F12/3C(FIX)+CABS method was implemented into both Psi4 and Quax. The use of automatic differentiation to achieve the nuclear and electric field derivatives was presented by utilizing Quax, which relies on the Python AD library JAX. The quality of the AD computations was assessed using both the aug-cc-pVXZ and cc-pVXZ-F12 (X = D, T, Q, 5) basis set families for the 16-molecule test set: H_2_O, H_2_S, CH_2_O, NH_3_, HNO, HOF, HCN, HNC, HCP, HBS, HF, HCl, CO, SiO, PN, and CS. Similar to absolute energies, the F12 correction was shown to reduce the basis set error for: (a) geometry optimizations, (b) dipole moments, (c) vibrational frequencies, and (d) IR intensities.

For the geometry optimizations, the minimal geometry obtained using AD gradients matched those computed by finite differences of energies to within 10^–6^ Angstrom and less than 10^–3^ degree. Although the aVDZ and VDZ-F12 series both provided geometries similar to conventional quadruple-ζ quality, MP2-F12/VDZ-F12 had a slightly smaller MAD and MSD than MP2-F12/aVDZ when compared to the MP2/aV[TQ5, Q5]Z limit. The convergence behavior of the two basis sets aligns with observations in past studies of MP2-F12 energies.

For the dipole moments, we observe that the AD implementation has higher accuracy than using the analytic relaxed 1-RDM. This suggests that caution should be taken if using the 1-RDM to compute MP2-F12 properties. The application of AD enables the F12 correction to align closely with finite difference computations, achieving agreement to within approximately 0.000001 a.u. However, the numerical precision of AD ultimately sets a limit on the accuracy achievable. In contrast to absolute energies and nuclear gradients, the aVXZ series provides more accurate dipole moments than VXZ-F12. Due to the consistency of the aVXZ family, it is highly recommended to use these basis sets for F12 properties. Following previous work, it is suggested to use the CABS singles correction for aVDZ and VXZ-F12 computations. Overall, the MP2-F12/aVXZ reduces the basis set incompleteness error significantly for μ_*z*_, achieving results similar to conventional (X + 2)-ζ quality.

For the IR intensities, MP2-F12/aVDZ converged slower to the complete basis set limit than for the nuclear gradients and dipole moments, with the intensities and frequencies falling between conventional aVTZ and aVQZ quality. The AD does not match as closely with the numerical results for the Hessian as well with about a 10^–5^ difference. This is thought to be due to the instability of degenerate eigenvalues in the AD process, which is a longstanding problem in algorithmic differentiation. It is notable that the frequencies still matched between AD and finite differences of energies to about 10^–2^ cm^–1^.

Automatic differentiation is rapidly growing in interest and has promising applications for scientific computing. From this work, we have shown that AD can be used for a highly sophisticated wave function method, explicitly correlated MP2, but not without some caveats. It is of high interest for the chemical community that continued research toward automatic differentiation with repeated eigenvalues take place in the computational sciences. This would be especially important for AD to be applied to multireference methods and excited-state methods.

## Supporting Information Available

Clarification on F12 integral types, optimized geometry parameters of all methods, dipole moments of all methods, mean average errors for AD and analytic dipole moment contributions compared to numerical results, figures for dipole moment basis set convergence, vibrational frequencies and IR intensities of all methods, and IR spectra for MP2-F12/3C(FIX)+CABS (PDF)
