# Supplementary material for: Automatic Differentiation for Explicitly Correlated MP2
Source: J Chem Theory Comput. 2024 Sep 23;20(19):8529–38. doi: 10.1021/acs.jctc.4c00818 (PMC11465469; doi:10.1021/acs.jctc.4c00818)
Supplement: Supplementary file 1 — ct4c00818_si_001.pdf [file ct4c00818_si_001.pdf]

Automatic Differentiation for Explicitly Correlated MP2

Erica C. Mitchell [†,‡], Justin M. Turney [‡], Henry F. Schaefer III [†,‡]

[†] Department of Chemistry, University of Georgia, 302 East Campus Road, Athens, Georgia 30602, United States

[‡] Center for Computational Quantum Chemistry, University of Georgia, 1004 Cedar Street, Athens, Georgia 30602, United States

Table of Contents

1 Methods 2
2 Geometry Optimizations 3
2.1 H_2O 3
2.2 H_2S 3
2.3 CH_2O 4
2.4 NH_3 5
2.5 HNO 5
2.6 HOF 6
2.7 HCN 6
2.8 HNC 7
2.9 HCP 7
2.10 HBS 8
2.11 HF 8
2.12 HCl 8
2.13 CO 9
2.14 SiO 9
2.15 PN 9
2.16 CS 10
3 Multipole Moments 11
4 IR Intensities 30
4.1 Vibrational Frequencies 30
4.2 IR Intensities 31
4.3 IR Spectra 32

1 Methods

**Integral Type**	**Notation**
Two-body Coulomb	$g^{p'q'}_{r's'}$
Contracted Gaussian Geminal	$f^{p'q'}_{r's'}$
Contracted Gaussian Geminal Squared	$(f^2)^{p'q'}_{r's'}$
Contracted Gaussian Geminal Times Coulomb	$(gf)^{p'q'}_{r's'}$
Gradient Norm of Contracted Gaussian Geminal	$\left\langle p'q' \middle| \left[\hat{f}_{12}\left[\hat{T}_{12},\ \hat{f}_{12}\right]\right] \middle| r's' \right\rangle$

Table S1. Integral types that are present in explicitly correlated methods.

2 Geometry Optimizations

The following tables present the equilibrium geometry parameters for the 16-molecule test set.

"R" indicates bond lengths, "B" indicates bond angles, "O" indicates dihedral angles, and "L" and "l" represent linear dihedral angles.

2.1 H_2O

Method	MP2-F12				MP2			
Basis	VDZ-F12		aVDZ		aVDZ	aVTZ	aVQZ	CBS
Program	Quax	Psi4	Quax	Psi4	Psi4			
R(1,2)	0.9589	0.9589	0.9602	0.9602	0.9658	0.9613	0.9588	0.9579
R(1,3)	0.9589	0.9589	0.9602	0.9602	0.9658	0.9613	0.9588	0.9579
B(2,1,3)	104.304	104.304	104.296	104.296	103.670	104.127	104.262	104.419

Table S2. Optimized geometry parameters for H_2O at various levels of theory computed with AD (Quax) and FINDIF of energies (Psi4). All bond lengths in Angstrom and angles in degrees.

2.2 H_2S

Method	MP2-F12				MP2			
Basis	VDZ-F12		aVDZ		aVDZ	aVTZ	aVQZ	CBS
Program	Quax	Psi4	Quax	Psi4	Psi4			
R(1,2)	1.3324	1.3324	1.3335	1.3335	1.3442	1.3336	1.3327	1.3323
R(1,3)	1.3324	1.3324	1.3335	1.3335	1.3442	1.3336	1.3327	1.3323
B(2,1,3)	92.217	92.217	92.304	92.304	92.303	92.147	92.214	92.217

Table S3. Optimized geometry parameters for H_2S at various levels of theory computed with AD (Quax) and FINDIF of energies (Psi4). All bond lengths in Angstrom and angles in degrees.

2.3 CH_2O

Method	MP2-F12				MP2			
Basis	VDZ-F12		aVDZ		aVDZ	aVTZ	aVQZ	CBS
Program	Quax	Psi4	Quax	Psi4	Psi4			
R(1,2)	1.2222	1.2128	1.2103	1.2103	1.2222	1.2128	1.2091	1.2081
R(2,3)	1.1111	1.1001	1.1007	1.1007	1.1111	1.1001	1.0990	1.0987
R(2,4)	1.1111	1.1001	1.1007	1.1007	1.1111	1.1001	1.0990	1.0987
B(1,2,3)	121.651	121.684	121.662	121.662	121.651	121.684	121.702	121.624
B(1,2,4)	121.651	121.684	121.662	121.662	121.651	121.684	121.702	121.624
B(3,2,4)	116.698	116.633	116.676	116.676	116.698	116.633	116.597	116.752
O(1,2,3,4)	0.002	0.000	0.000	0.000	0.002	0.000	0.000	0.000
O(3,2,1,4)	-0.002	0.000	0.000	0.000	-0.002	0.000	0.000	0.000
O(4,2,1,3)	0.002	0.000	0.000	0.000	0.002	0.000	0.000	0.000

Table S4. Optimized geometry parameters for CH_2O at various levels of theory computed with AD (Quax) and FINDIF of energies (Psi4). All bond lengths in Angstrom and angles in degrees.

2.4 NH_3

Method	MP2-F12				MP2			
Basis	VDZ-F12		aVDZ		aVDZ	aVTZ	aVQZ	CBS
Program	Quax	Psi4	Quax	Psi4	Psi4			
R(1,2)	1.0099	1.0099	1.0112	1.0112	1.0203	1.0118	1.0100	1.0092
R(1,3)	1.0099	1.0099	1.0112	1.0112	1.0203	1.0118	1.0100	1.0092
R(1,4)	1.0099	1.0099	1.0112	1.0112	1.0203	1.0118	1.0100	1.0092
B(2,1,3)	106.956	106.956	106.890	106.891	106.031	106.818	106.888	106.997
B(2,1,4)	106.956	106.956	106.891	106.891	106.031	106.818	106.888	106.997
B(3,1,4)	106.956	106.956	106.891	106.891	106.031	106.818	106.888	106.997
O(2,1,3,4)	60.656	60.656	60.802	60.802	62.675	60.963	60.808	60.565
O(3,1,2,4)	-60.656	-60.656	-60.802	-60.802	-62.675	-60.963	-60.808	-60.565
O(4,1,2,3)	60.656	60.656	60.802	60.802	62.675	60.963	60.808	60.565

Table S5. Optimized geometry parameters for NH_3 at various levels of theory computed with AD (Quax) and FINDIF of energies (Psi4). All bond lengths in Angstrom and angles in degrees.

2.5 HNO

Method	MP2-F12				MP2			
Basis	VDZ-F12		aVDZ		aVDZ	aVTZ	aVQZ	CBS
Program	Quax	Psi4	Quax	Psi4	Psi4			
R(1,2)	1.0496	1.0496	1.0509	1.0509	1.0591	1.0505	1.0490	1.0486
R(2,3)	1.2174	1.2174	1.2190	1.2190	1.2322	1.2216	1.2173	1.2164
B(1,2,3)	107.743	107.742	107.728	107.728	107.338	107.665	107.784	107.764

Table S6. Optimized geometry parameters for HNO at various levels of theory computed with AD (Quax) and FINDIF of energies (Psi4). All bond lengths in Angstrom and angles in degrees.

2.6 HOF

Method	MP2-F12				MP2			
Basis	VDZ-F12		aVDZ		aVDZ	aVTZ	aVQZ	CBS
Program	Quax	Psi4	Quax	Psi4	Psi4			
R(1,2)	0.9679	0.9679	0.9691	0.9691	0.9746	0.9700	0.9673	0.9668
R(2,3)	1.4238	1.4238	1.4235	1.4235	1.4453	1.4282	1.4237	1.4212
B(2,1,3)	97.997	97.997	98.070	98.070	97.412	97.914	97.993	98.061

Table S7. Optimized geometry parameters for HOF at various levels of theory computed with AD (Quax) and FINDIF of energies (Psi4). All bond lengths in Angstrom and angles in degrees.

2.7 HCN

Method	MP2-F12				MP2			
Basis	VDZ-F12		aVDZ		aVDZ	aVTZ	aVQZ	CBS
Program	Quax	Psi4	Quax	Psi4	Psi4			
R(1,2)	1.0644	1.0645	1.0660	1.0660	1.0774	1.0651	1.0643	1.0639
R(1,3)	1.1628	1.1628	1.1646	1.1646	1.1818	1.1666	1.1636	1.1622
L(2,1,3)	180.000	180.000	180.000	180.000	180.000	180.000	180.000	180.000
l(2,1,3)	180.000	180.000	180.000	180.000	180.000	180.000	180.000	180.000

Table S8. Optimized geometry parameters for HCN at various levels of theory computed with AD (Quax) and FINDIF of energies (Psi4). All bond lengths in Angstrom and angles in degrees.

2.8 HNC

Method	MP2-F12				MP2			
Basis	VDZ-F12		aVDZ		aVDZ	aVTZ	aVQZ	CBS
Program	Quax	Psi4	Quax	Psi4	Psi4			
R(1,2)	1.1727	1.1727	1.1749	1.1749	1.1914	1.1762	1.1733	1.1722
R(2,3)	0.9968	0.9968	0.9983	0.9983	1.0062	0.9976	0.9962	0.9963
L(1,2,3)	180.000	180.000	180.000	180.000	180.000	180.000	180.000	180.000
l(1,2,3)	180.000	180.000	180.000	180.000	180.000	180.000	180.000	180.000

Table S9. Optimized geometry parameters for HNC at various levels of theory computed with AD (Quax) and FINDIF of energies (Psi4). All bond lengths in Angstrom and angles in degrees.

2.9 HCP

Method	MP2-F12				MP2			
Basis	VDZ-F12		aVDZ		aVDZ	aVTZ	aVQZ	CBS
Program	Quax	Psi4	Quax	Psi4	Psi4			
R(1,2)	1.0714	1.0714	1.1749	1.1749	1.0849	1.0726	1.0713	1.0713
R(2,3)	1.5491	1.5491	0.9983	0.9983	1.5702	1.5555	1.5516	1.5492
L(2,1,3)	180.000	180.000	180.000	180.000	180.000	180.000	180.000	180.000
l(2,1,3)	180.000	180.000	180.000	180.000	180.000	180.000	180.000	180.000

Table S10. Optimized geometry parameters for HCP at various levels of theory computed with AD (Quax) and FINDIF of energies (Psi4). All bond lengths in Angstrom and angles in degrees.

2.10 HBS

Method	MP2-F12				MP2			
Basis	VDZ-F12		aVDZ		aVDZ	aVTZ	aVQZ	CBS
Program	Quax	Psi4	Quax	Psi4	Psi4			
R(1,2)	1.1690	1.1690	1.1703	1.1704	1.1805	1.1695	1.1688	1.1685
R(1,3)	1.5995	1.5995	1.6017	1.6018	1.6206	1.6053	1.6019	1.5997
L(2,1,3)	180.000	180.000	180.000	180.000	180.000	180.000	180.000	180.000
l(2,1,3)	180.000	180.000	180.000	180.000	180.000	180.000	180.000	180.000

Table S11. Optimized geometry parameters for HBS at various levels of theory computed with AD (Quax) and FINDIF of energies (Psi4). All bond lengths in Angstrom and angles in degrees.

2.11 HF

Method	MP2-F12				MP2			
Basis	VDZ-F12		aVDZ		aVDZ	aVTZ	aVQZ	CBS
Program	Quax	Psi4	Quax	Psi4	Psi4			
R(1,2)	0.9191	0.9191	0.9204	0.9204	0.9242	0.9213	0.9188	0.9180

Table S12. Optimized geometry parameters for HF at various levels of theory computed with AD (Quax) and FINDIF of energies (Psi4). All bond lengths in Angstrom and angles in degrees.

2.12 HCl

Method	MP2-F12				MP2			
Basis	VDZ-F12		aVDZ		aVDZ	aVTZ	aVQZ	CBS
Program	Quax	Psi4	Quax	Psi4	Psi4			
R(1,2)	1.2719	1.2719	1.2730	1.2730	1.2824	1.2725	1.2719	1.2717

Table S13. Optimized geometry parameters for HCl at various levels of theory computed with AD (Quax) and FINDIF of energies (Psi4). All bond lengths in Angstrom and angles in degrees.

2.13 CO

Method	MP2-F12				MP2			
Basis	VDZ-F12		aVDZ		aVDZ	aVTZ	aVQZ	CBS
Program	Quax	Psi4	Quax	Psi4	Psi4			
R(1,2)	1.1346	1.1346	1.1364	1.1364	1.1489	1.1388	1.1349	1.1337

Table S14. Optimized geometry parameters for CO at various levels of theory computed with AD (Quax) and FINDIF of energies (Psi4). All bond lengths in Angstrom and angles in degrees.

2.14 SiO

Method	MP2-F12				MP2			
Basis	VDZ-F12		aVDZ		aVDZ	aVTZ	aVQZ	CBS
Program	Quax	Psi4	Quax	Psi4	Psi4			
R(1,2)	1.5245	1.5245	1.5240	1.5240	1.5502	1.5305	1.5264	1.5233

Table S15. Optimized geometry parameters for SiO at various levels of theory computed with AD (Quax) and FINDIF of energies (Psi4). All bond lengths in Angstrom and angles in degrees.

2.15 PN

Method	MP2-F12				MP2			
Basis	VDZ-F12		aVDZ		aVDZ	aVTZ	aVQZ	CBS
Program	Quax	Psi4	Quax	Psi4	Psi4			
R(1,2)	1.5143	1.5143	1.5150	1.5150	1.5378	1.5227	1.5170	1.5140

Table S16. Optimized geometry parameters for PN at various levels of theory computed with AD (Quax) and FINDIF of energies (Psi4). All bond lengths in Angstrom and angles in degrees.

2.16 CS

Method	MP2-F12				MP2			
Basis	VDZ-F12		aVDZ		aVDZ	aVTZ	aVQZ	CBS
Program	Quax	Psi4	Quax	Psi4	Psi4			
R(1,2)	1.5335	1.5335	1.5349	1.5349	1.5545	1.5392	1.5354	1.5331

Table S17. Optimized geometry parameters for CS at various levels of theory computed with AD (Quax) and FINDIF of energies (Psi4). All bond lengths in Angstrom and angles in degrees.

3 Multipole Moments

Method	MP2-F12		MP2				
Basis	VDZ-F12		VDZ-F12	VTZ-F12	VQZ-F12	V5Z-F12	CBS (aVXZ)
Program	Quax	Psi4	Psi4				
H_2O	-0.75622	-0.75623	-0.75836	-0.74254	-0.74025	-0.73986	-0.73967
H_2S	-0.42671	-0.42671	-0.41549	-0.40160	-0.39887	-0.39770	-0.39774
CH_2O	-0.94667	-0.94668	-0.93422	-0.93818	-0.94251	-0.94520	-0.94727
NH_3	-0.64145	-0.64146	-0.64712	-0.62776	-0.62474	-0.62383	-0.62356
HNO	0.00000	0.00000	0.00000	0.00000	0.00000	0.00000	0.00000
HOF	0.00000	0.00000	0.00000	0.00000	0.00000	0.00000	0.00000
HCN	-1.19665	-1.19666	-1.18681	-1.19177	-1.19460	-1.19633	-1.19712
HNC	1.28644	1.28646	1.28859	1.28326	1.28258	1.28225	1.28256
HCP	-0.18316	-0.18317	-0.17704	-0.18094	-0.18258	-0.18403	-0.18496
HBS	-0.53873	-0.53873	-0.52074	-0.53132	-0.53805	-0.54218	-0.54561
HF	-0.71958	-0.71959	-0.72075	-0.71276	-0.71183	-0.71231	-0.71285
HCl	-0.46377	-0.46378	-0.45659	-0.44276	-0.44183	-0.44191	-0.44194
CO	0.10227	0.10227	0.10981	0.10296	0.10103	0.09942	0.09921
SiO	1.17877	1.17877	1.17121	1.18534	1.18795	1.18945	1.18992
PN	1.05404	1.05405	1.04545	1.04985	1.05422	1.05541	1.05623
CS	0.87766	0.87768	0.88492	0.87757	0.87569	0.87373	0.87224

Table S18. Dipole moments (μ_z) of the test set for the VXZ-F12 basis set family computed with AD (Quax) and FINDIF of energies (Psi4). Note that the MP2/CBS limit was done using the aVXZ series. Units in a.u.

Method	MP2-F12		MP2				
Basis	aVDZ		aVDZ	aVTZ	aVQZ	aV5Z	CBS
Program	Quax	Psi4	Psi4				
H_2O	-0.74016	-0.74016	-0.73781	-0.73093	-0.73538	-0.73766	-0.73967
H_2S	-0.40771	-0.40771	-0.40445	-0.38777	-0.39309	-0.39521	-0.39774
CH_2O	-0.94658	-0.94659	-0.93078	-0.93570	-0.94151	-0.94440	-0.94727
NH_3	-0.62600	-0.62600	-0.62094	-0.61928	-0.62165	-0.62260	-0.62356
HNO	0.00000	0.00000	0.00000	0.00000	0.00000	0.00000	0.00000
HOF	0.00000	0.00000	0.00000	0.00000	0.00000	0.00000	0.00000
HCN	-1.19923	-1.19924	-1.19074	-1.19207	-1.19427	-1.19567	-1.19712
HNC	1.28429	1.28430	1.29277	1.28187	1.28139	1.28196	1.28256
HCP	-0.18336	-0.18337	-0.19510	-0.17972	-0.18301	-0.18372	-0.18496
HBS	-0.54953	-0.54952	-0.53873	-0.53108	-0.53906	-0.54204	-0.54561
HF	-0.71088	-0.71088	-0.70803	-0.70730	-0.70958	-0.71124	-0.71285
HCl	-0.44955	-0.44955	-0.45196	-0.43329	-0.43825	-0.43992	-0.44194
CO	0.10220	0.10220	0.11063	0.10361	0.10108	0.10011	0.09921
SiO	1.17692	1.17694	1.15495	1.18119	1.18694	1.18861	1.18992
PN	1.05800	1.05803	1.03517	1.05061	1.05292	1.05487	1.05623
CS	0.87192	0.87194	0.87145	0.87800	0.87443	0.87365	0.87224

Table S19. Dipole moments (μ_z) of the test set for the aVXZ basis set family computed with AD (Quax) and FINDIF of energies (Psi4). Units in a.u.

Method	MP2-F12					
Basis	VDZ-F12			aVDZ		
Program	Quax	Psi4	MPQC	Quax	Psi4	MPQC
H_2O	-0.75622	-0.75623	-0.75588	-0.74016	-0.74016	-0.74041
H_2S	-0.42671	-0.42671	-0.42713	-0.40771	-0.40771	-0.40771
CH_2O	-0.94667	-0.94668	-0.94616	-0.94658	-0.94659	-0.94771
NH_3	-0.64145	-0.64146	-0.64138	-0.62600	-0.62600	-0.62603
HNO	0.00000	0.00000	0.00000	0.00000	0.00000	0.00000
HOF	0.00000	0.00000	0.00000	0.00000	0.00000	0.00000
HCN	-1.19665	-1.19666	-1.19647	-1.19923	-1.19924	-1.19946
HNC	1.28644	1.28646	1.28671	1.28429	1.28430	1.28325
HCP	-0.18316	-0.18317	-0.18486	-0.18336	-0.18337	-0.18118
HBS	-0.53873	-0.53873	-0.53934	-0.54953	-0.54952	-0.54996
HF	-0.71958	-0.71959	-0.71924	-0.71088	-0.71088	-0.71125
HCl	-0.46377	-0.46378	-0.46374	-0.44955	-0.44955	-0.45031
CO	0.10227	0.10227	0.10296	0.10220	0.10220	0.10077
SiO	1.17877	1.17877	1.17678	1.17692	1.17694	1.18045
PN	1.05404	1.05405	1.05261	1.05800	1.05803	1.06050
CS	0.87766	0.87768	0.87718	0.87192	0.87194	0.87068

Table S18. Dipole moments (μ_z) of the test set at various levels of theory computed with AD (Quax), FINDIF of energies (Psi4), and analytic relaxed 1-RDMs (MPQC). Units in a.u.

Method	HF		MP2		S2		F12	
Program	Quax	MPQC	Quax	MPQC	Quax	MPQC	Quax	MPQC
H_2O	3.4E-09	9.3E-10	2.3E-06	8.7E-10	1.9E-08	2.1E-06	3.4E-07	3.5E-04
H_2S	1.2E-09	4.4E-09	2.7E-07	7.9E-09	6.8E-08	4.7E-06	8.9E-08	4.2E-04
CH_2O	1.2E-08	2.2E-09	1.2E-05	8.0E-09	1.2E-07	1.4E-06	5.7E-07	5.2E-04
NH_3	1.2E-08	5.1E-10	2.4E-07	3.0E-10	3.4E-07	1.1E-04	9.3E-07	1.9E-04
HNO	0.0E+00	0.0E+00	0.0E+00	0.0E+00	2.0E-10	2.0E-09	0.0E+00	0.0E+00
HOF	0.0E+00	0.0E+00	0.0E+00	0.0E+00	0.0E+00	4.5E-09	0.0E+00	0.0E+00
HCN	2.8E-09	3.5E-10	4.8E-06	3.5E-10	7.8E-08	8.8E-07	7.4E-08	1.9E-04
HNC	1.9E-09	6.9E-10	1.8E-05	1.7E-09	2.3E-08	2.1E-06	4.9E-07	2.5E-04
HCP	2.4E-09	2.1E-08	3.1E-06	6.5E-09	1.5E-07	5.1E-06	2.8E-07	1.7E-03
HBS	1.3E-08	2.4E-08	6.7E-06	4.4E-08	2.0E-07	5.7E-05	1.2E-07	5.5E-04
HF	5.0E-09	5.8E-10	3.0E-06	1.1E-09	1.7E-08	7.1E-06	3.2E-07	3.4E-04
HCl	7.0E-10	9.3E-09	9.6E-07	2.1E-09	4.2E-08	5.9E-06	2.5E-07	4.3E-05
CO	1.0E-10	1.9E-09	3.6E-07	3.5E-09	9.7E-09	5.9E-06	3.8E-08	6.8E-04
SiO	4.7E-09	8.1E-09	1.6E-06	4.6E-08	3.6E-07	2.9E-06	6.0E-07	2.0E-03
PN	4.0E-09	3.0E-08	1.7E-05	6.1E-09	2.1E-06	7.5E-07	3.7E-06	1.4E-03
CS	1.1E-08	1.9E-08	2.5E-05	6.1E-09	2.8E-07	1.9E-06	3.5E-07	5.0E-04

Table S19. MAE of the test set computed at MP2-F12/VDZ-F12 comparing AD (Quax) and analytic relaxed 1-RDMs (MPQC) to FINDIF of energies (Psi4), Units in a.u.

Method	HF		MP2		S2		F12	
Program	Quax	MPQC	Quax	MPQC	Quax	MPQC	Quax	MPQC
H_2O	3.60E-09	1.69E-10	3.14E-06	5.89E-10	6.79E-08	1.80E-06	1.55E-07	2.51E-04
H_2S	2.00E-10	3.96E-10	2.78E-07	1.46E-08	1.17E-07	1.62E-08	1.06E-07	4.95E-06
CH_2O	7.70E-09	8.98E-10	6.59E-06	1.61E-09	4.59E-08	9.77E-06	2.65E-07	1.11E-03
NH_3	1.00E-09	1.37E-09	5.40E-06	1.80E-09	1.96E-07	7.85E-06	2.25E-07	1.35E-05
HNO	0.00E+00	0.00E+00	0.00E+00	0.00E+00	0.00E+00	5.10E-10	1.30E-09	1.30E-09
HOF	0.00E+00	0.00E+00	0.00E+00	0.00E+00	0.00E+00	1.41E-09	1.30E-09	1.30E-09
HCN	2.40E-09	1.67E-09	4.07E-06	6.60E-11	6.65E-08	2.13E-06	1.19E-07	2.22E-04
HNC	3.90E-09	2.09E-09	8.69E-06	4.23E-10	1.58E-07	1.05E-05	3.45E-07	1.06E-03
HCP	1.20E-09	3.52E-09	3.16E-06	7.33E-09	1.25E-07	4.84E-06	2.09E-07	2.19E-03
HBS	1.43E-08	1.51E-10	1.34E-05	1.17E-08	5.96E-07	5.21E-06	7.87E-07	4.51E-04
HF	4.10E-09	7.37E-10	3.18E-06	8.78E-10	1.25E-07	2.05E-10	1.82E-07	3.69E-04
HCl	1.30E-09	1.78E-09	3.08E-07	1.18E-09	5.84E-08	3.09E-07	1.22E-07	7.55E-04
CO	9.00E-10	1.27E-09	1.41E-06	5.12E-09	5.84E-08	1.22E-05	6.00E-09	1.41E-03
SiO	1.80E-09	3.82E-09	1.93E-05	3.54E-08	4.80E-07	1.19E-05	1.80E-06	3.50E-03
PN	6.12E-08	4.05E-10	2.69E-05	7.46E-09	9.99E-06	5.00E-08	1.19E-05	2.47E-03
CS	1.24E-08	1.49E-10	2.32E-05	2.83E-10	3.14E-07	5.21E-09	8.17E-07	1.26E-03

Table S20. MSE of the test set computed at MP2-F12/aVDZ comparing AD (Quax) and analytic relaxed 1-RDMs (MPQC) to FINDIF of energies (Psi4), Units in a.u.

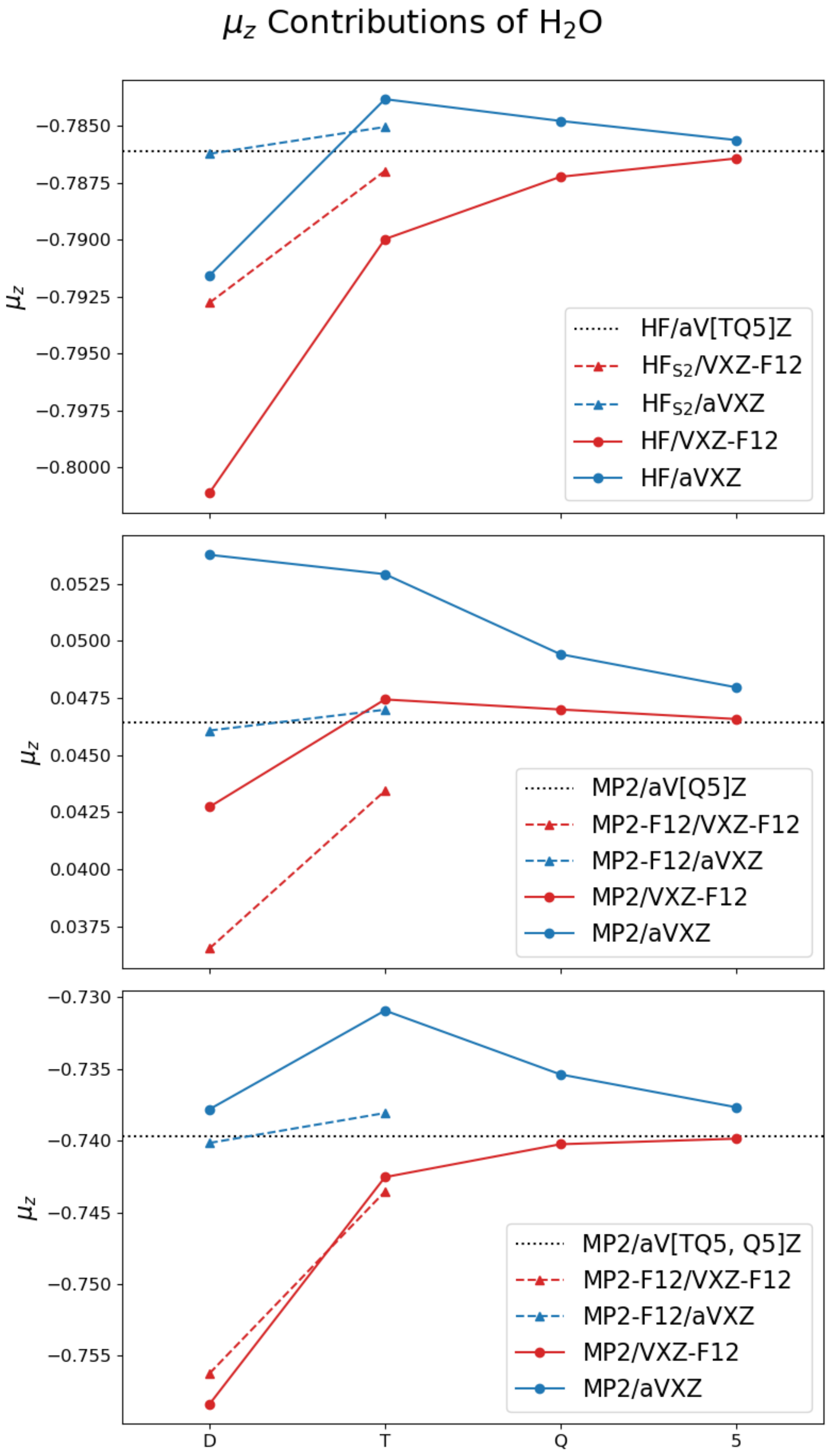

Fig S1. Dipole moment (μ_z) contributions of H_2O in a.u.

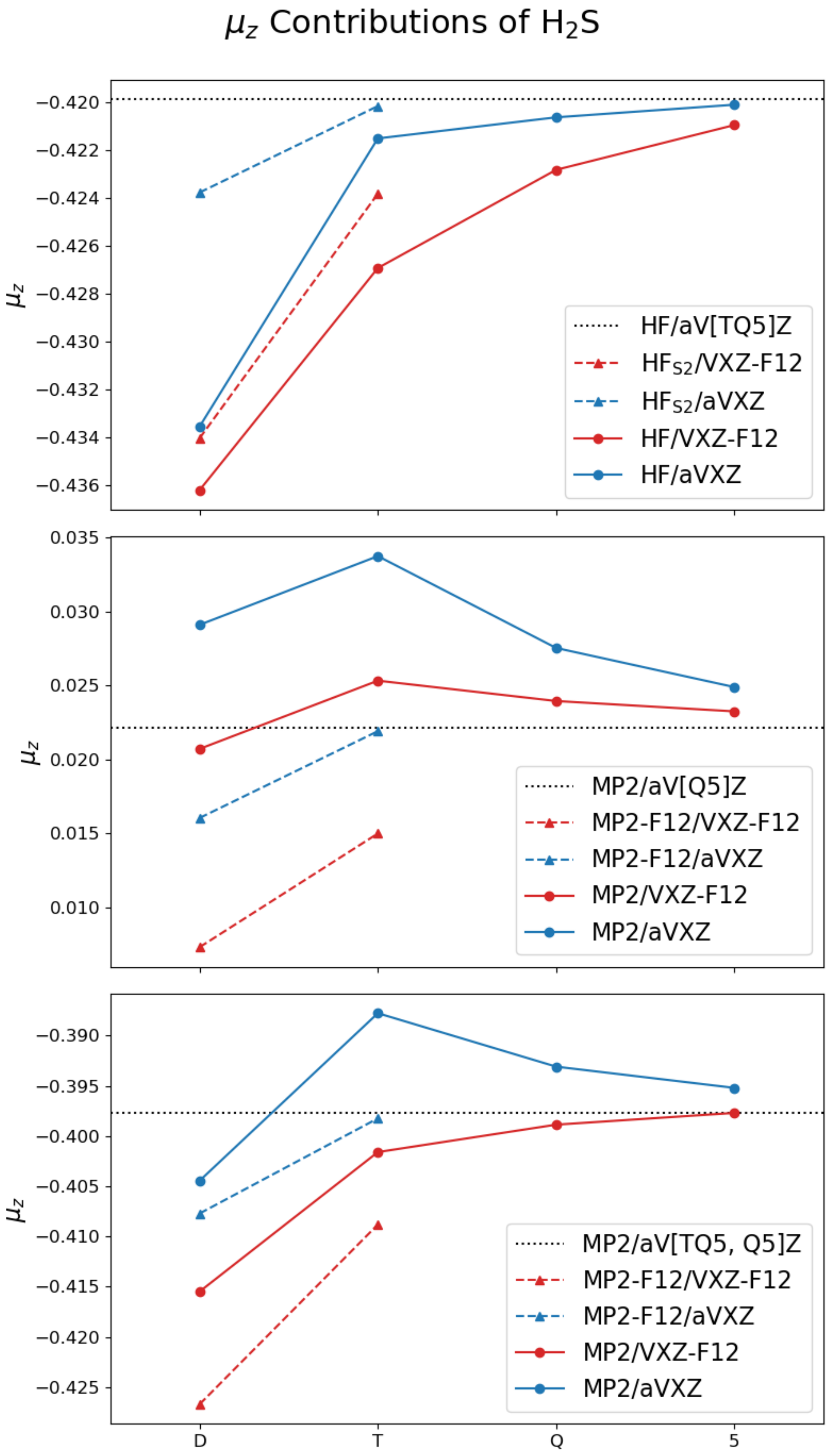

Fig S2. Dipole moment (μ_z) contributions of H_2S in a.u.

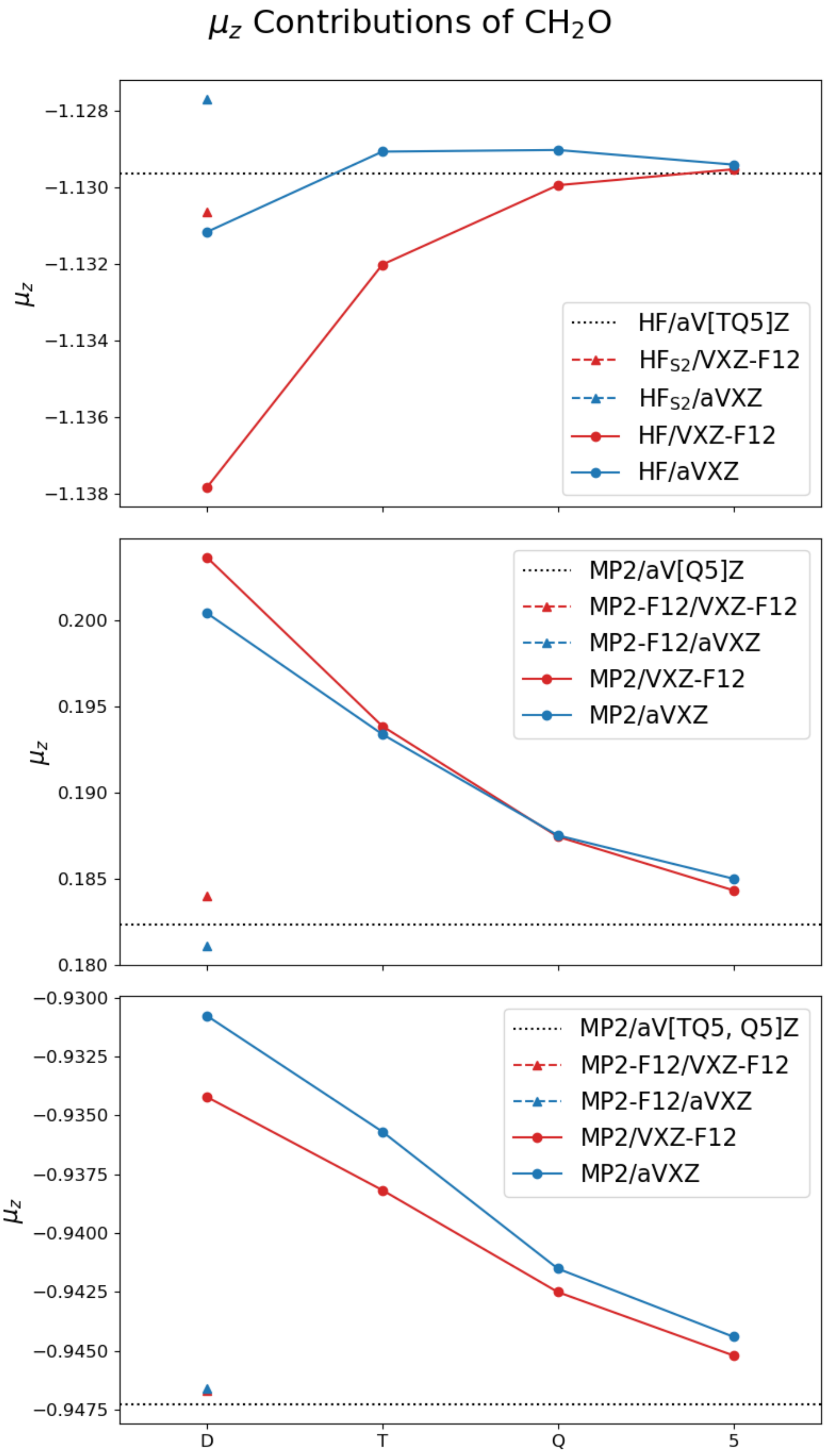

Fig S3. Dipole moment (μ_z) contributions of CH_2O in a.u.

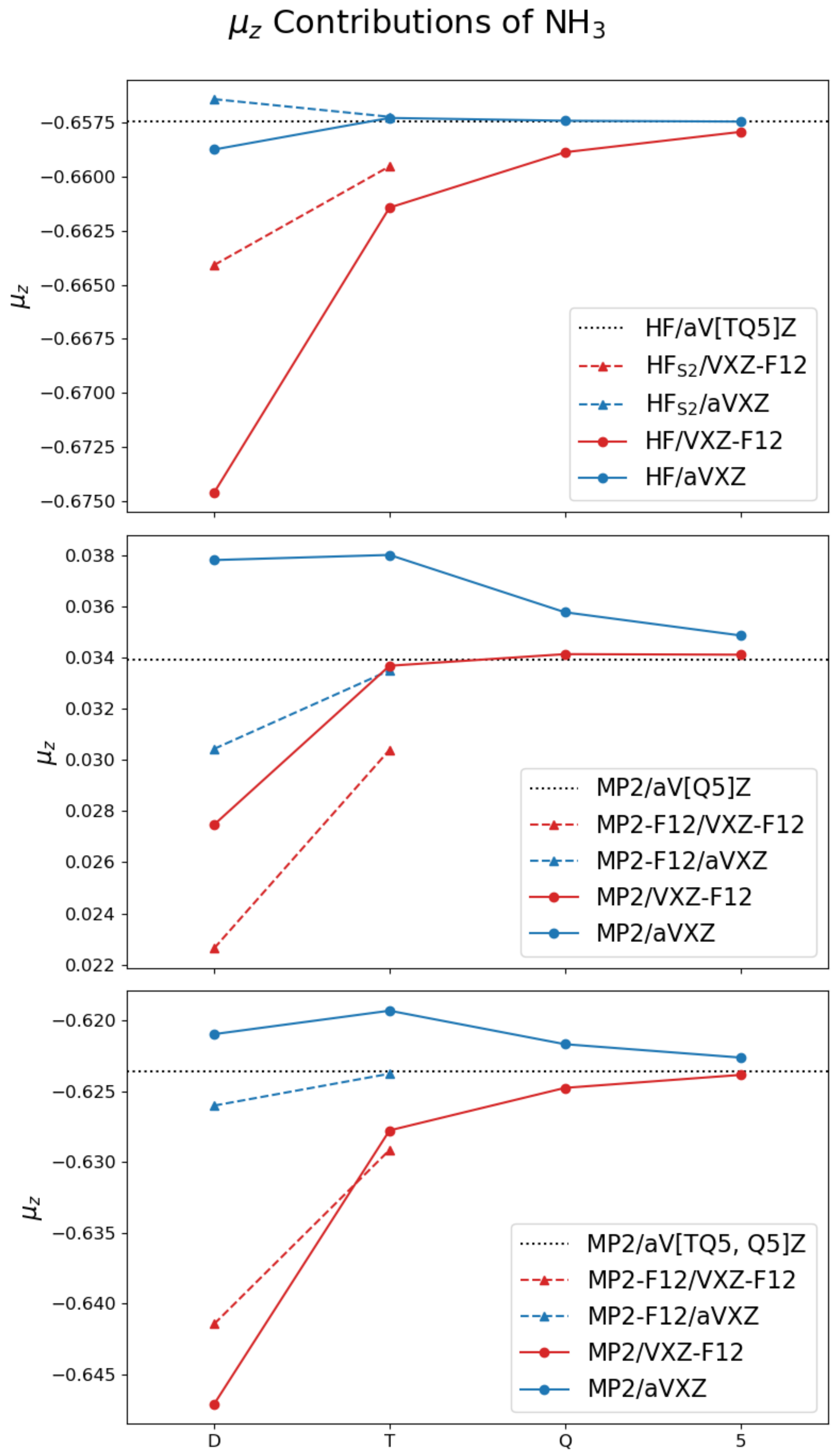

Fig S4. Dipole moment (μ_z) contributions of NH_3 in a.u.

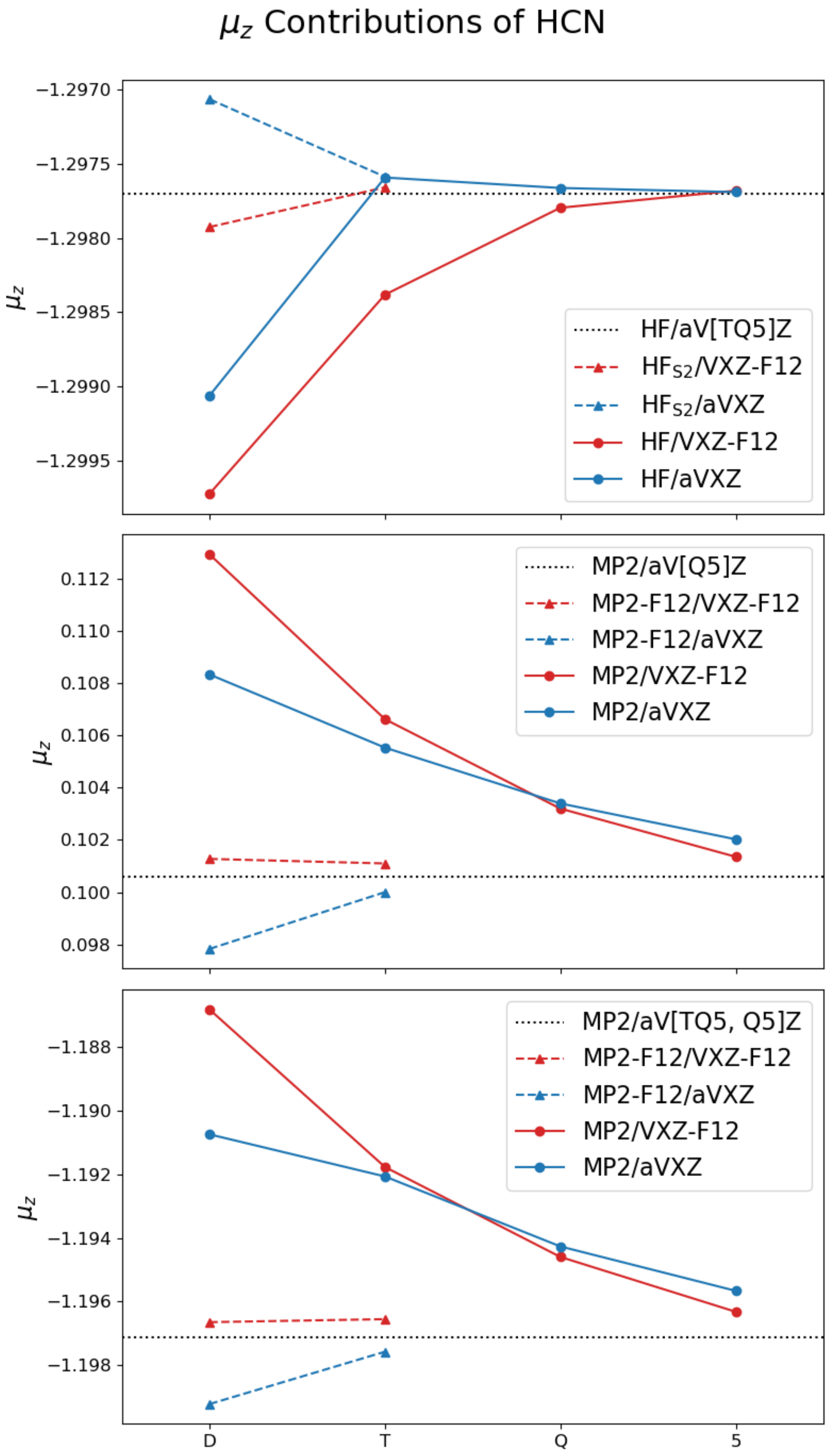

Fig S5. Dipole moment (μ_z) contributions of HCN in a.u.

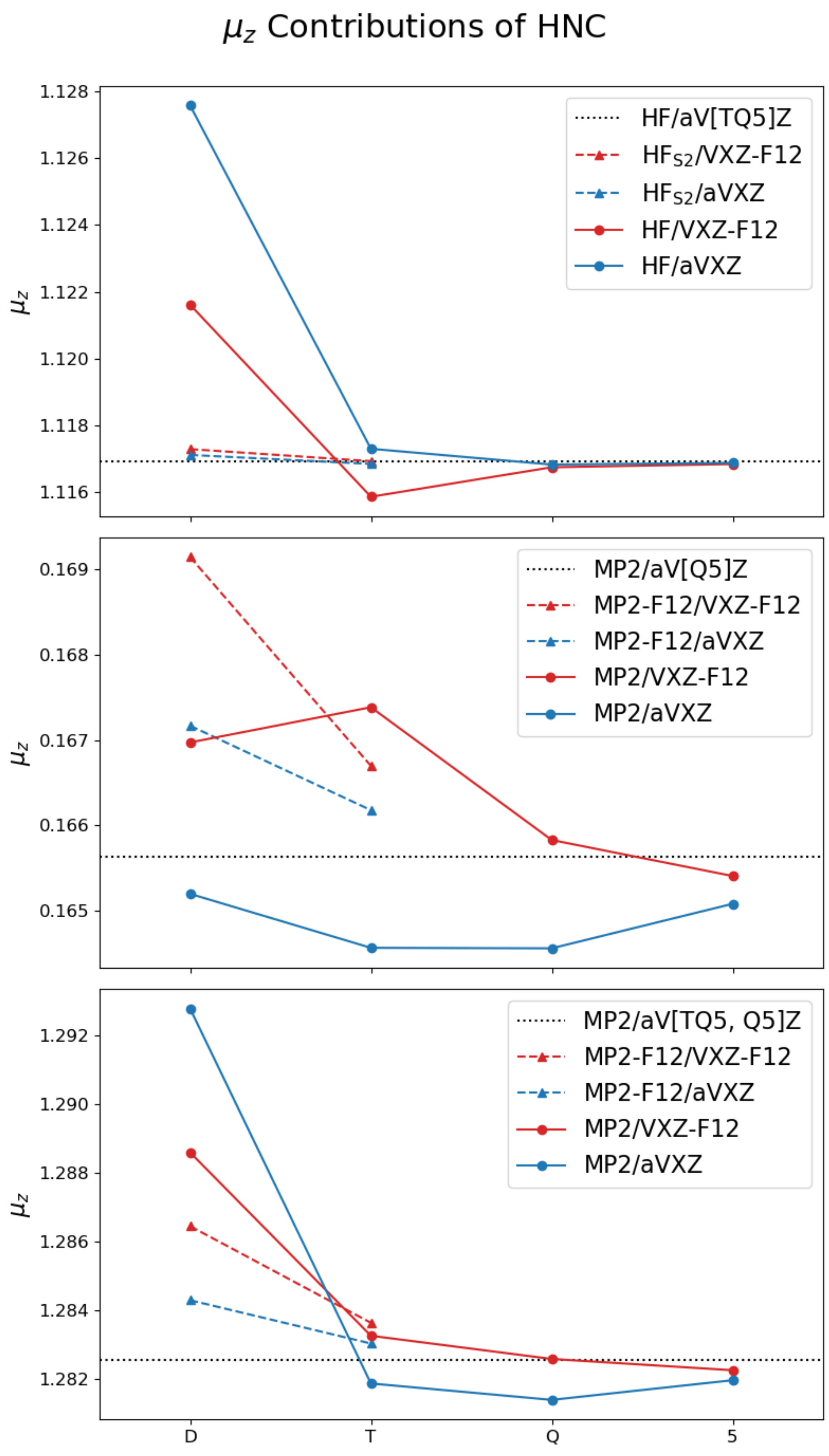

Fig S6. Dipole moment (μ_z) contributions of HNC in a.u.

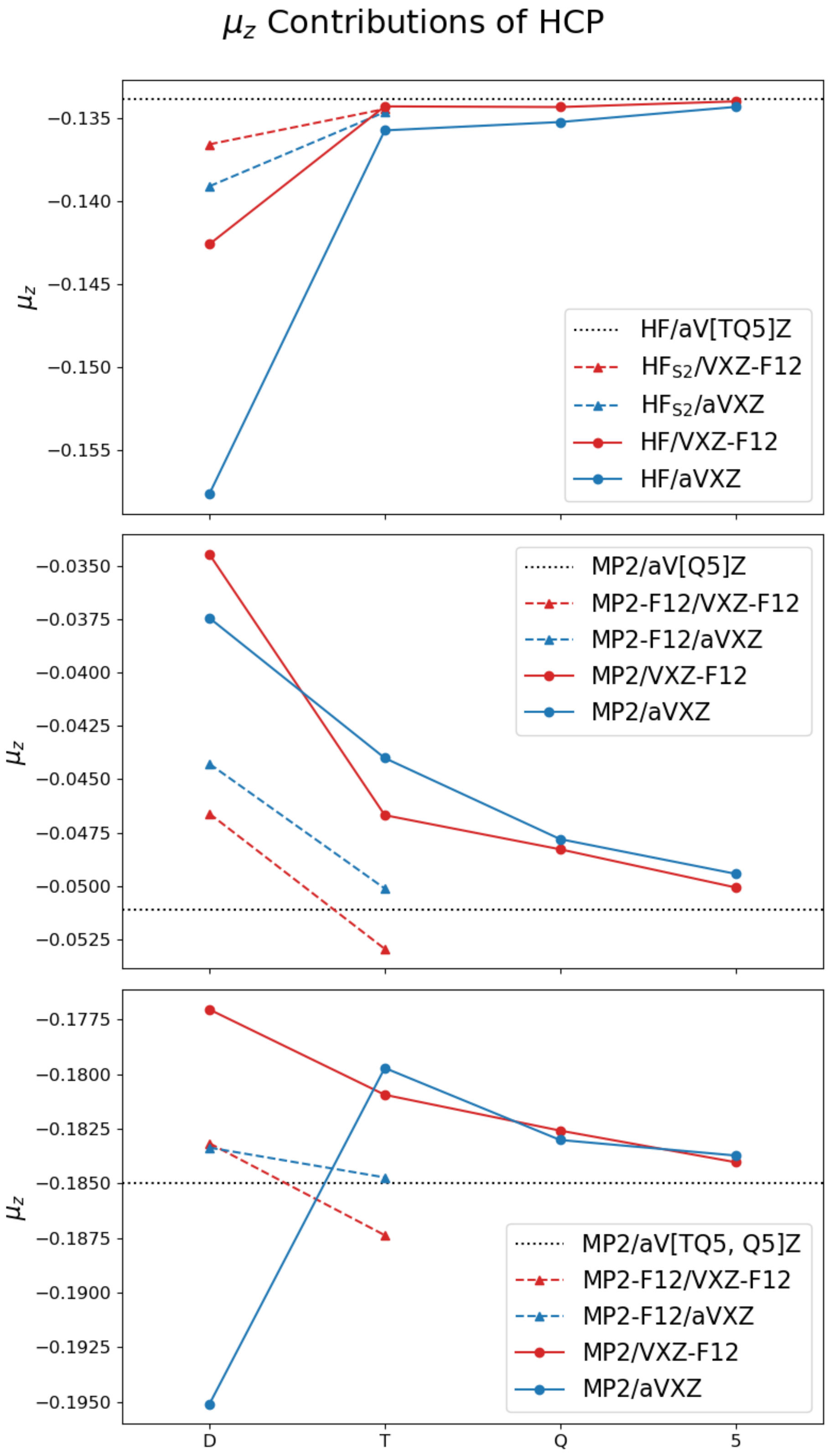

Fig S7. Dipole moment (μ_z) contributions of HCP in a.u.

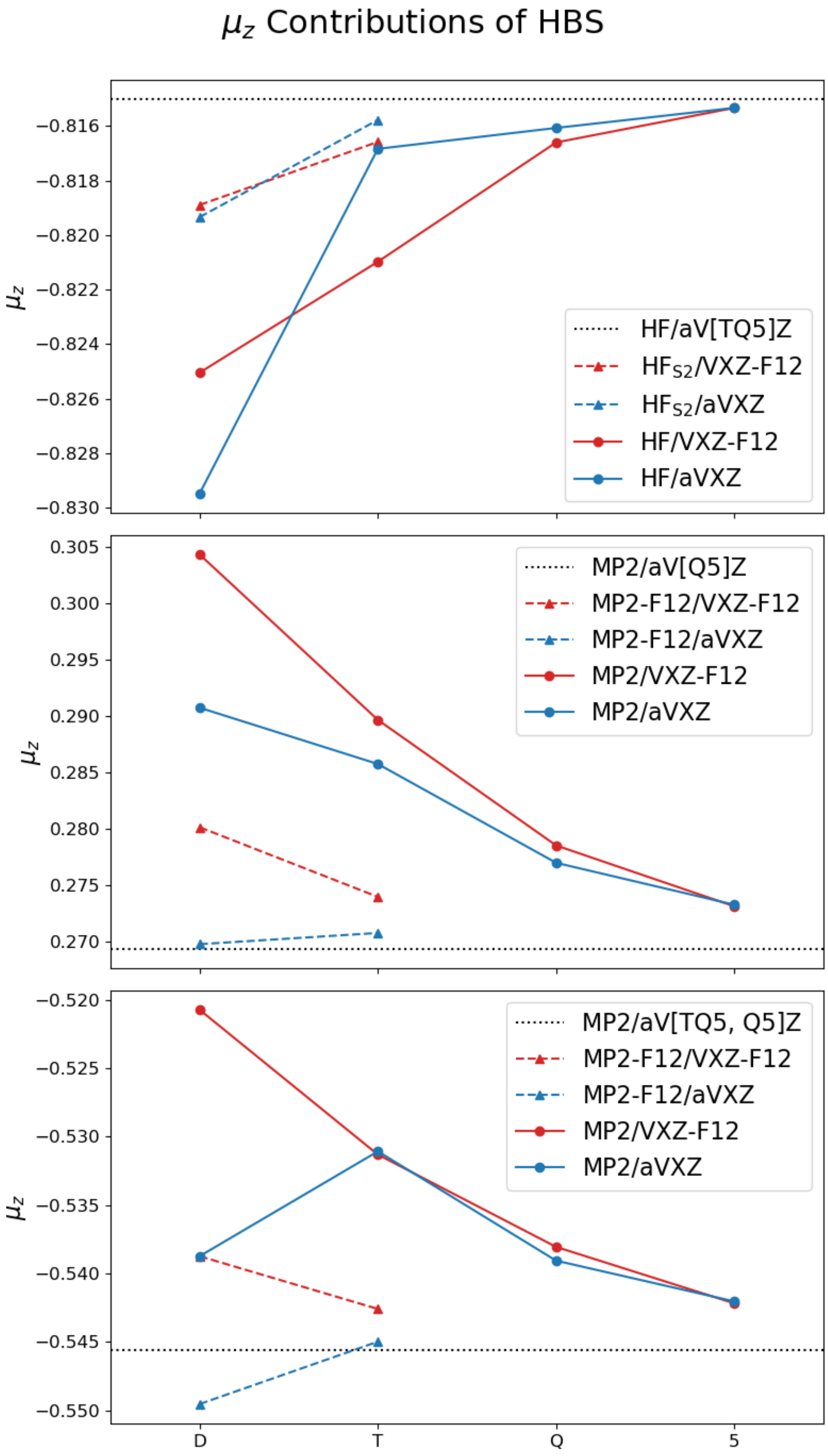

Fig S8. Dipole moment (μ_z) contributions of HBS in a.u.

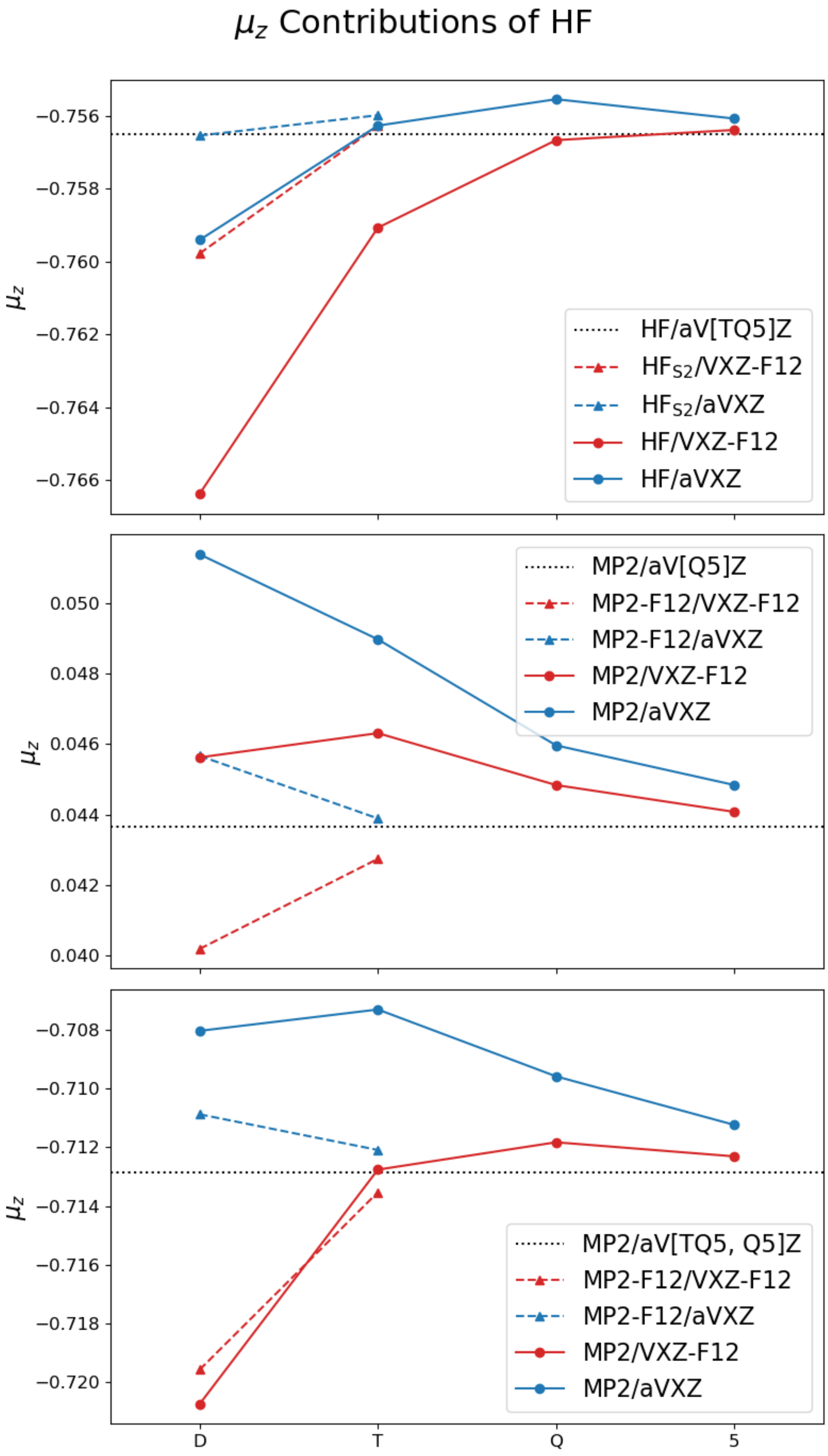

Fig S9. Dipole moment (μ_z) contributions of HF in a.u.

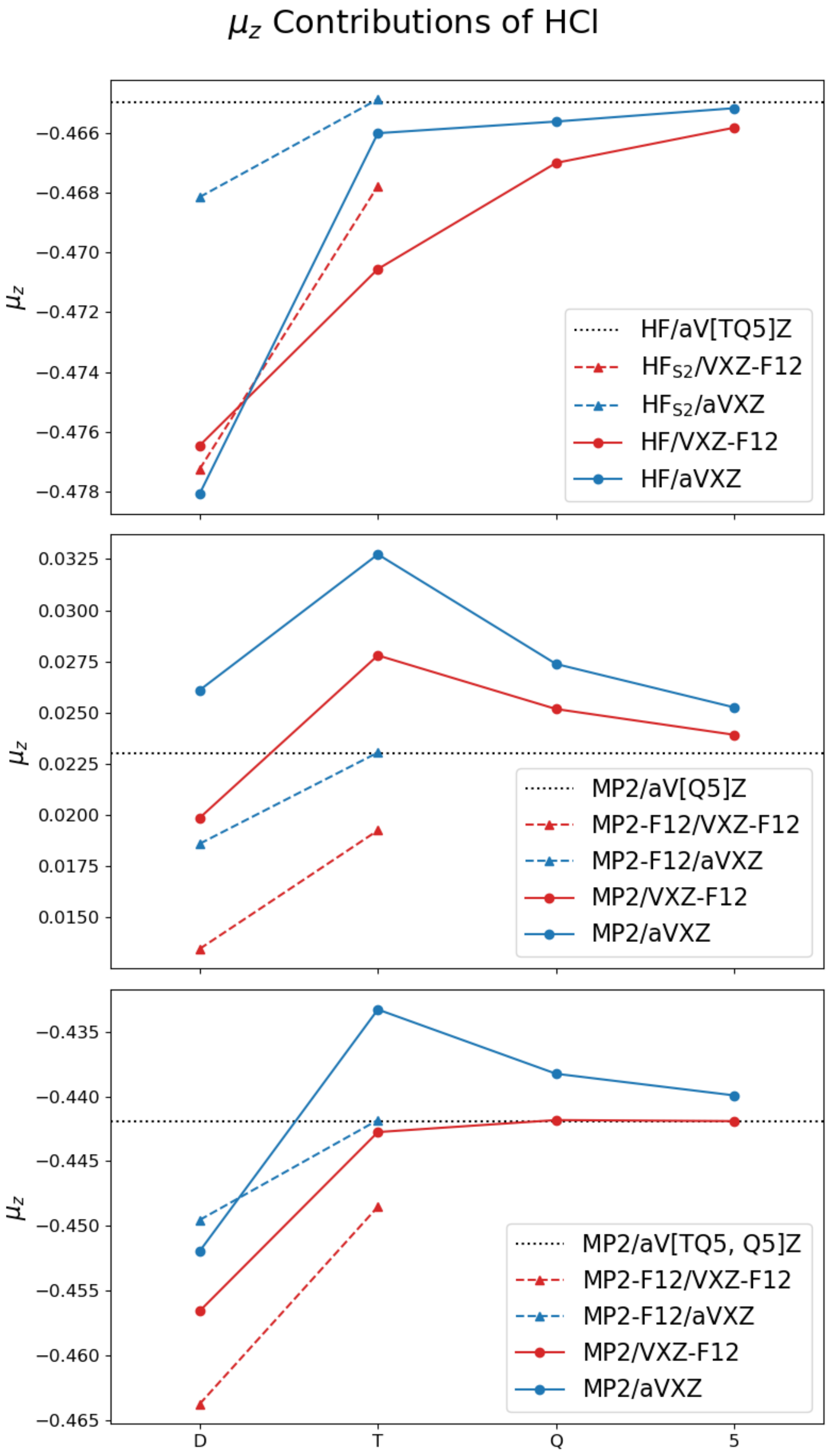

Fig S10. Dipole moment (μ_z) contributions of HCl in a.u.

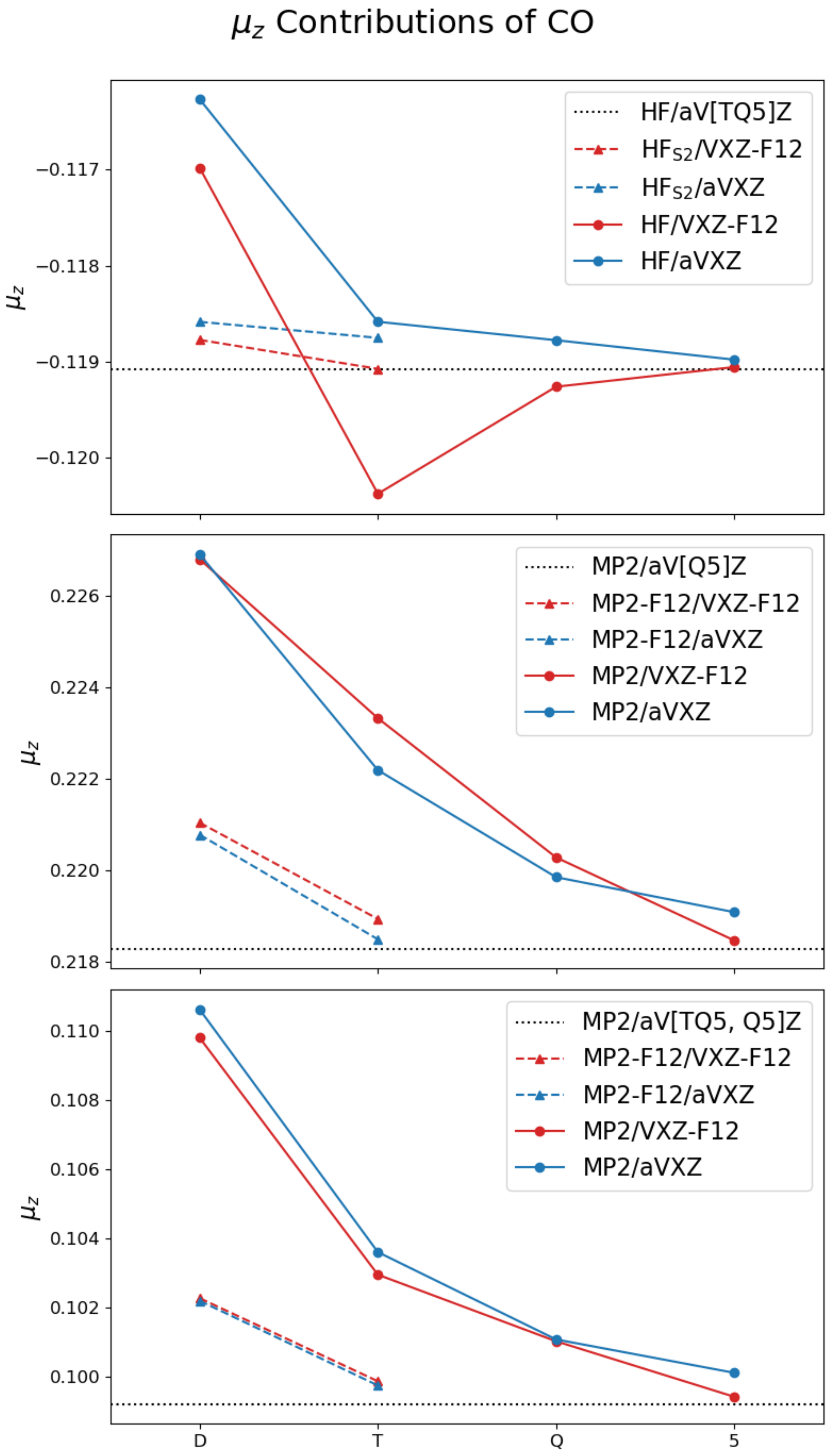

Fig S11. Dipole moment (μ_z) contributions of CO in a.u.

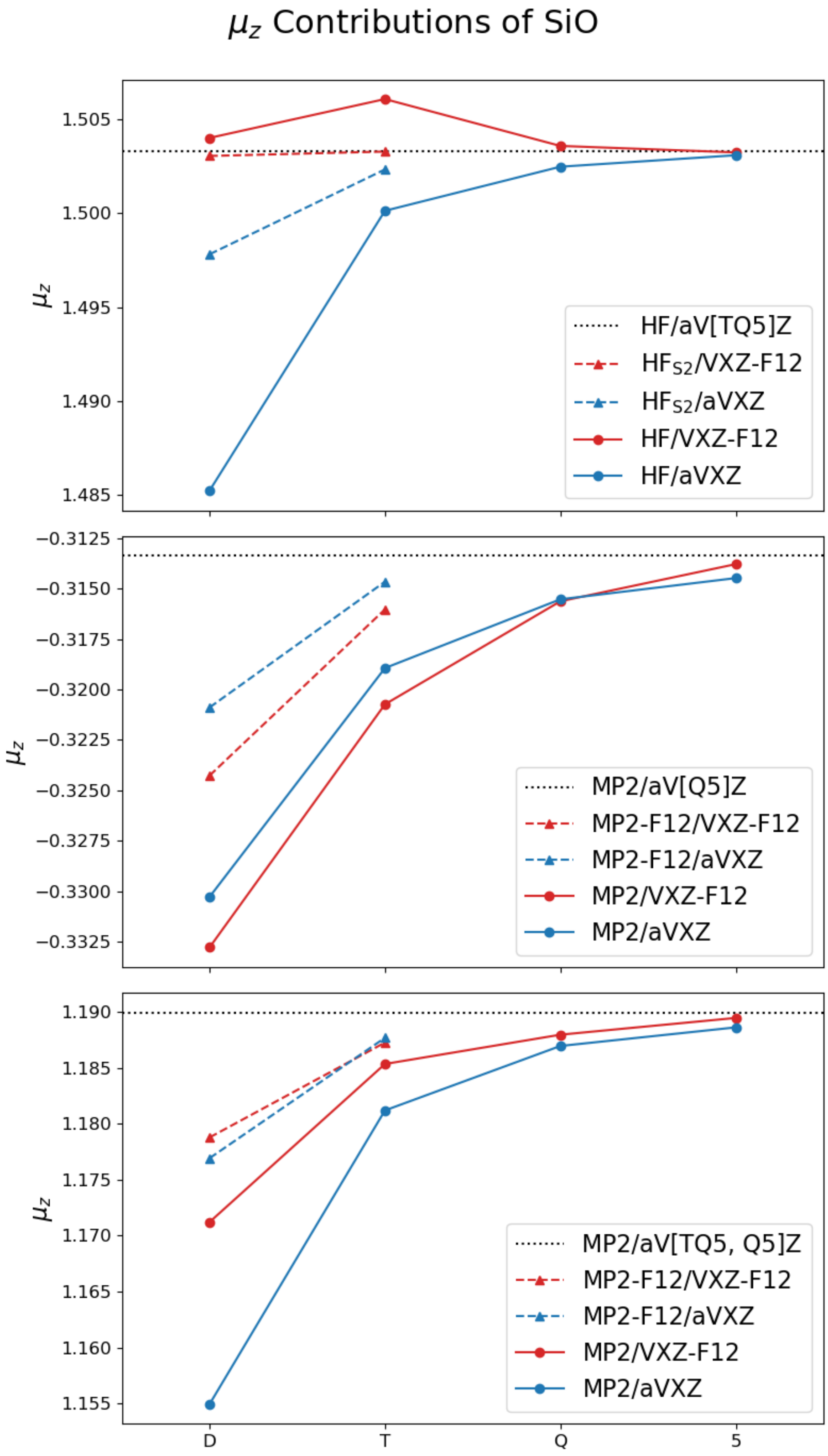

Fig S12. Dipole moment (μ_z) contributions of SiO in a.u.

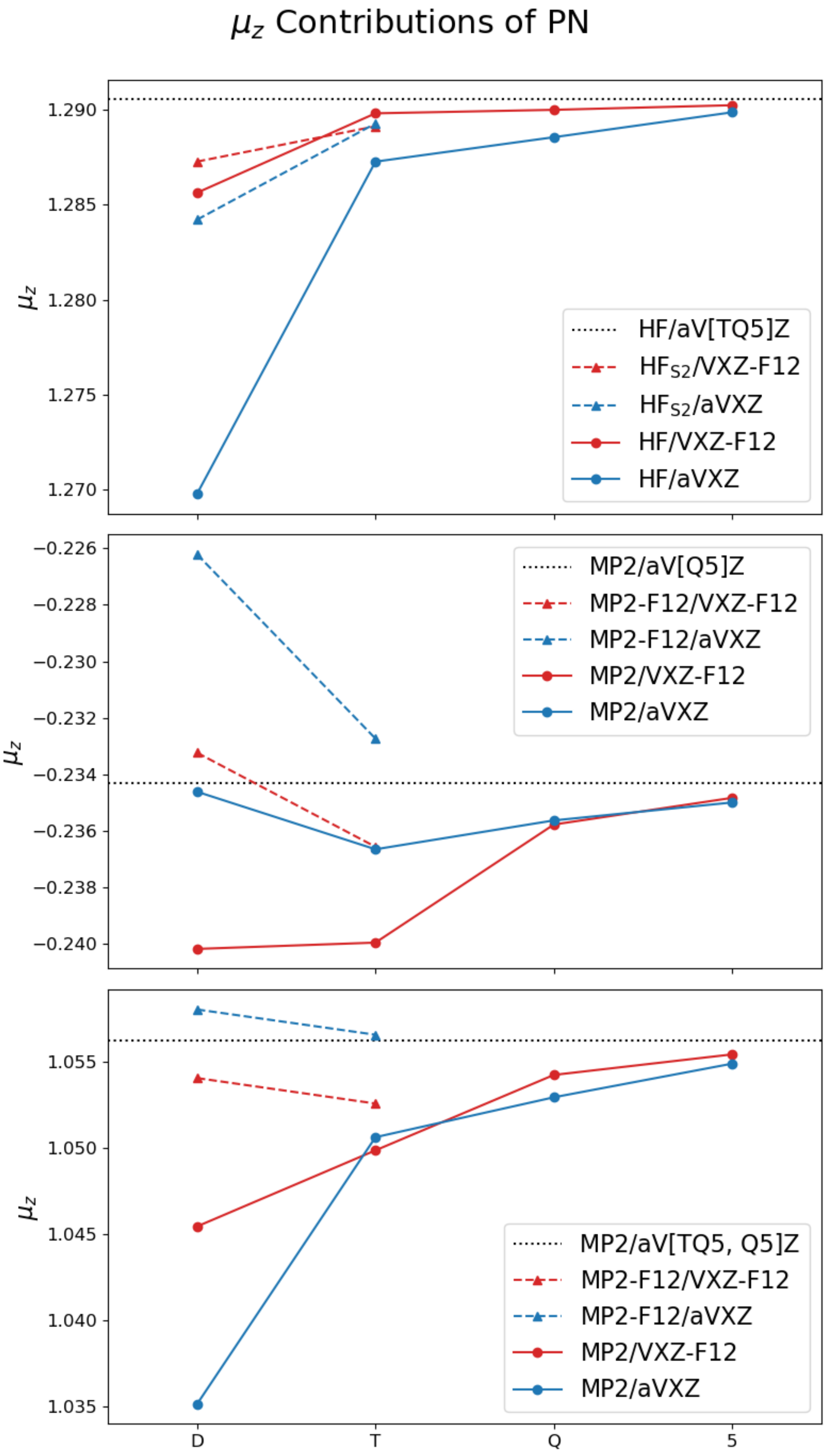

Fig S13. Dipole moment (μ_z) contributions of PN in a.u.

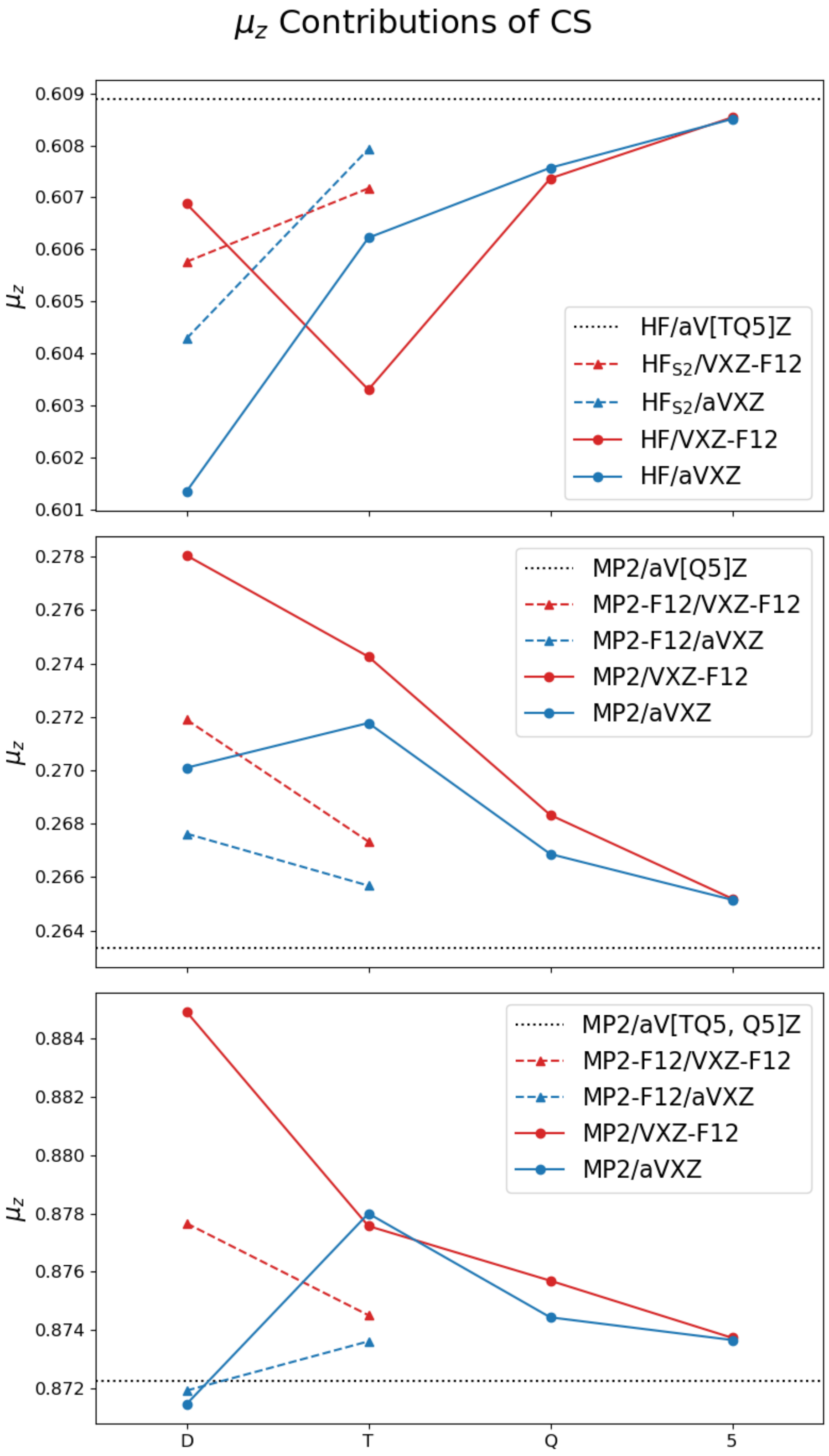

Fig S14. Dipole moment (μ_z) contributions of CS in a.u.

4 IR Intensities

4.1 Vibrational Frequencies

Program	Quax	Psi4	ORCA			
Method	MP2-F12 aVDZ	MP2-F12 aVDZ	MP2 aVDZ	MP2 aVTZ	MP2 aVQZ	MP2 aV5Z
H_2O	1625.60	1625.61	1605.01	1623.77	1635.06	1636.59
	3832.96	3832.98	3878.78	3836.10	3821.17	3817.88
	3958.99	3959.02	4015.37	3963.12	3947.13	3944.12
H_2S	1213.75	1213.73	1191.53	1216.61	1217.38	1217.81
	2779.39	2779.38	2843.59	2777.54	2775.87	2773.53
	2799.16	2799.32	2866.44	2796.77	2795.29	2792.76
HF	4128.71	4128.73	4150.74	4144.48	4111.99	4106.29
HCl	3043.76	3043.77	3119.87	3042.27	3037.99	3035.71
CO	2122.71	2122.70	2175.99	2128.73	2112.99	2109.52
SiO	1201.78	1201.82	1215.91	1207.72	1203.41	1200.48
PN	1211.96	1211.97	1239.69	1222.03	1211.56	1208.75
CS	1309.37	1309.56	1362.44	1313.97	1307.75	1305.41

Table S21. Harmonic vibrational frequencies of the test set computed with AD (Quax) and FINDIF of energies (Psi4 and ORCA). Units in cm^{-1}.

4.2 IR Intensities

Program	Quax	ORCA			
Method	MP2-F12 aVDZ	MP2 aVDZ	MP2 aVTZ	MP2 aVQZ	MP2 aV5Z
H_2O	72.77	68.54	72.04	73.08	73.57
	6.66	4.33	5.60	6.03	6.23
	81.25	68.97	76.23	78.37	79.21
H_2S	0.80	0.92	0.84	0.78	0.73
	0.49	0.00	0.17	0.35	0.44
	1.23	0.12	0.71	1.10	1.27
HF	126.61	115.68	120.47	122.59	123.59
HCl	57.56	46.14	51.83	55.07	56.40
CO	36.91	35.84	36.09	36.38	36.43
SiO	22.58	21.50	21.68	21.83	22.00
PN	9.19	8.33	8.98	9.06	9.11
CS	46.52	43.50	43.74	44.64	44.97

Table S22. IR intensities of the test set computed with AD (Quax), and analytic relaxed 1-RDMs with finite difference normal modes (ORCA). Units in km·mol^{-1}.

4.3 IR Spectra

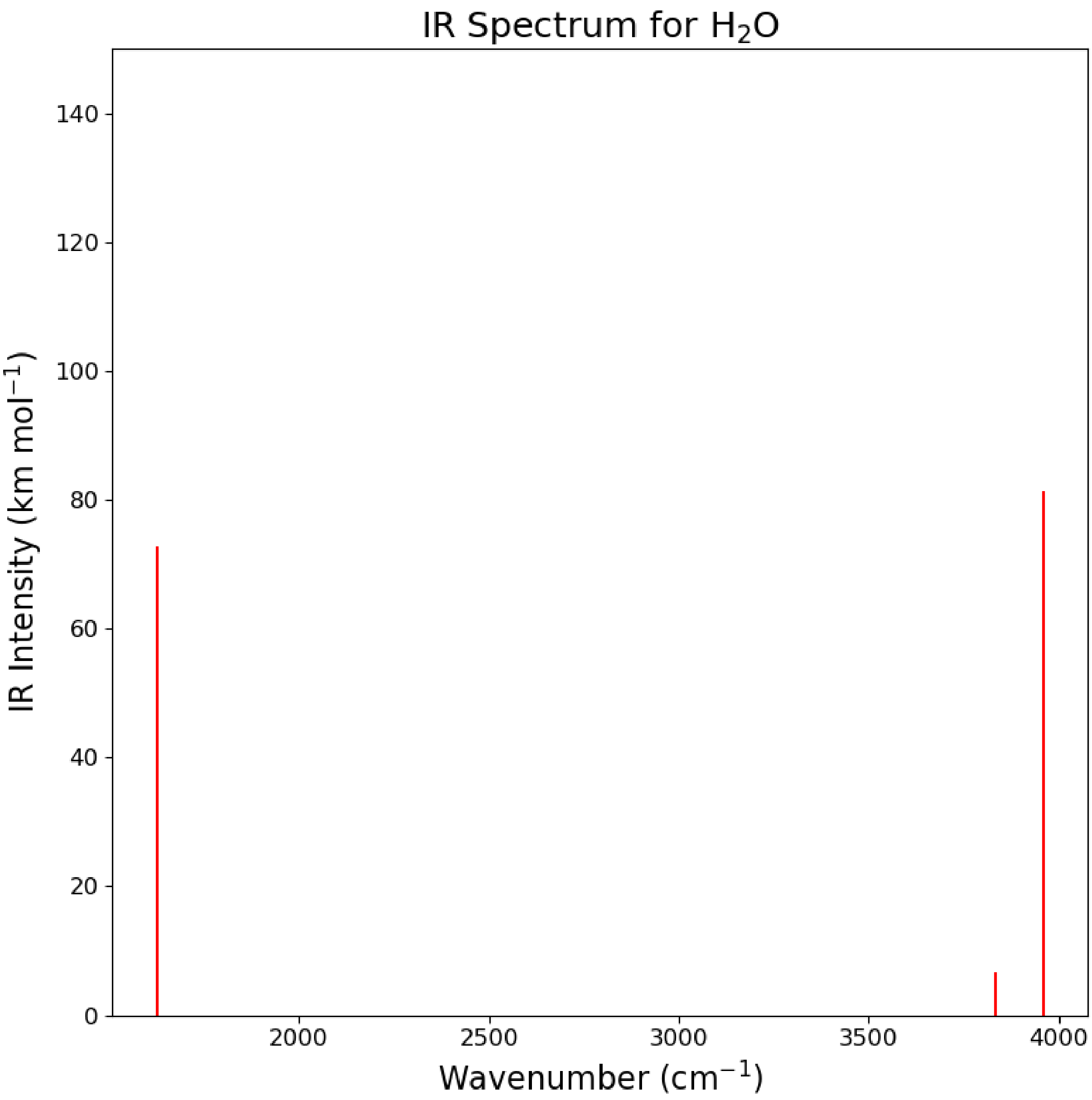

Fig S15. IR spectrum of H_2O at the MP2-F12/aVDZ level of theory computed using AD.

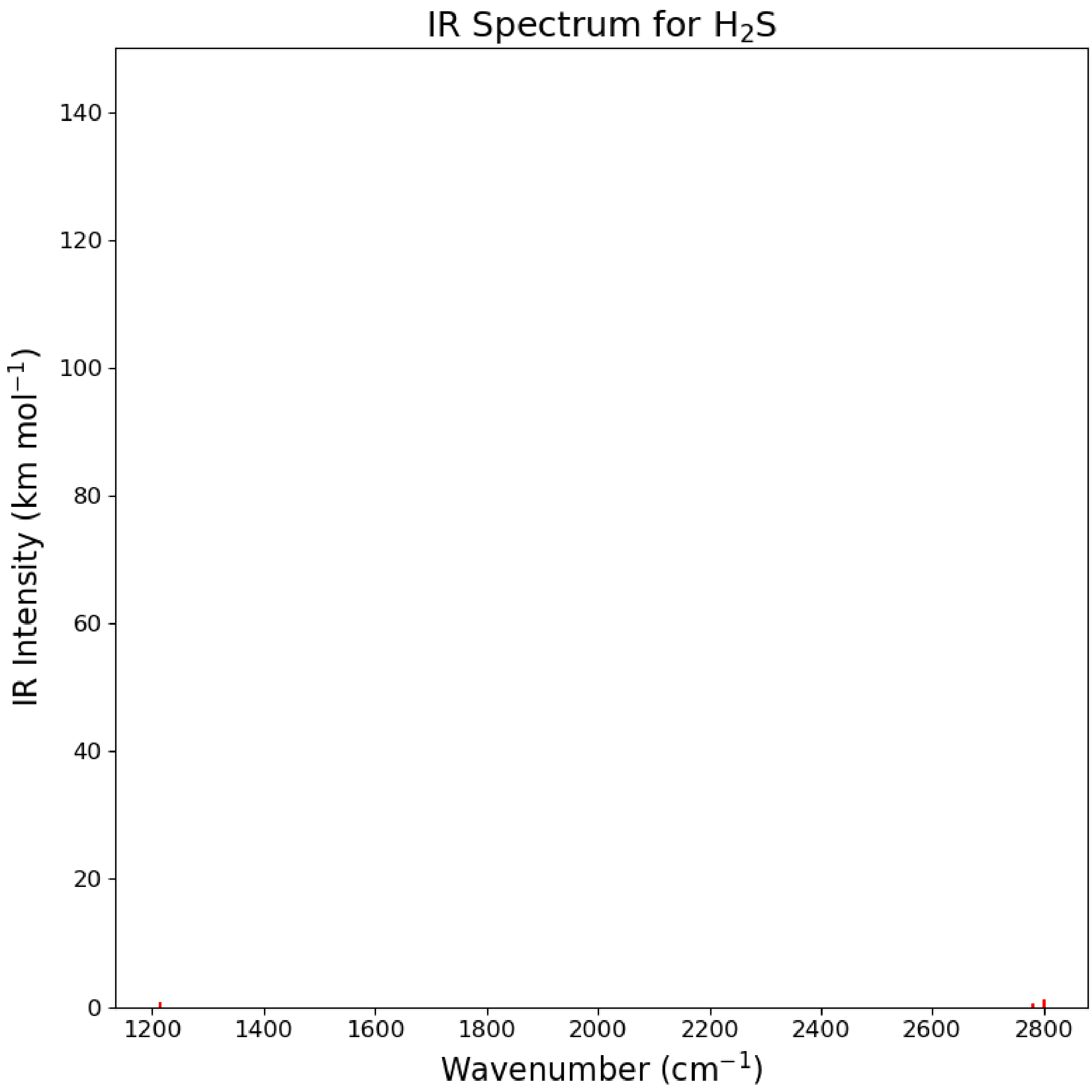

Fig S16. IR spectrum of H_2S at the MP2-F12/aVDZ level of theory computed using AD.

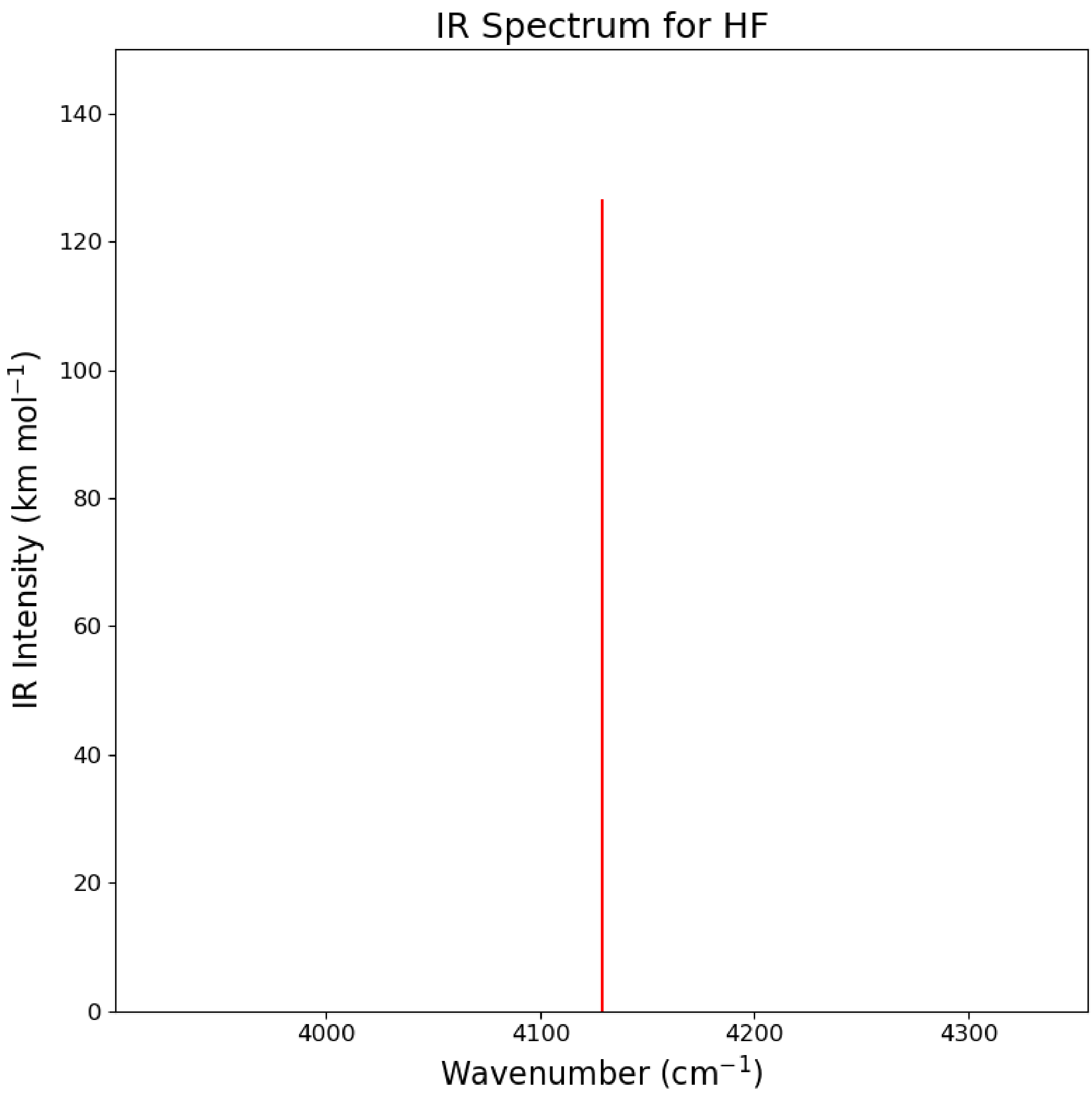

Fig S17. IR spectrum of HF at the MP2-F12/aVDZ level of theory computed using AD.

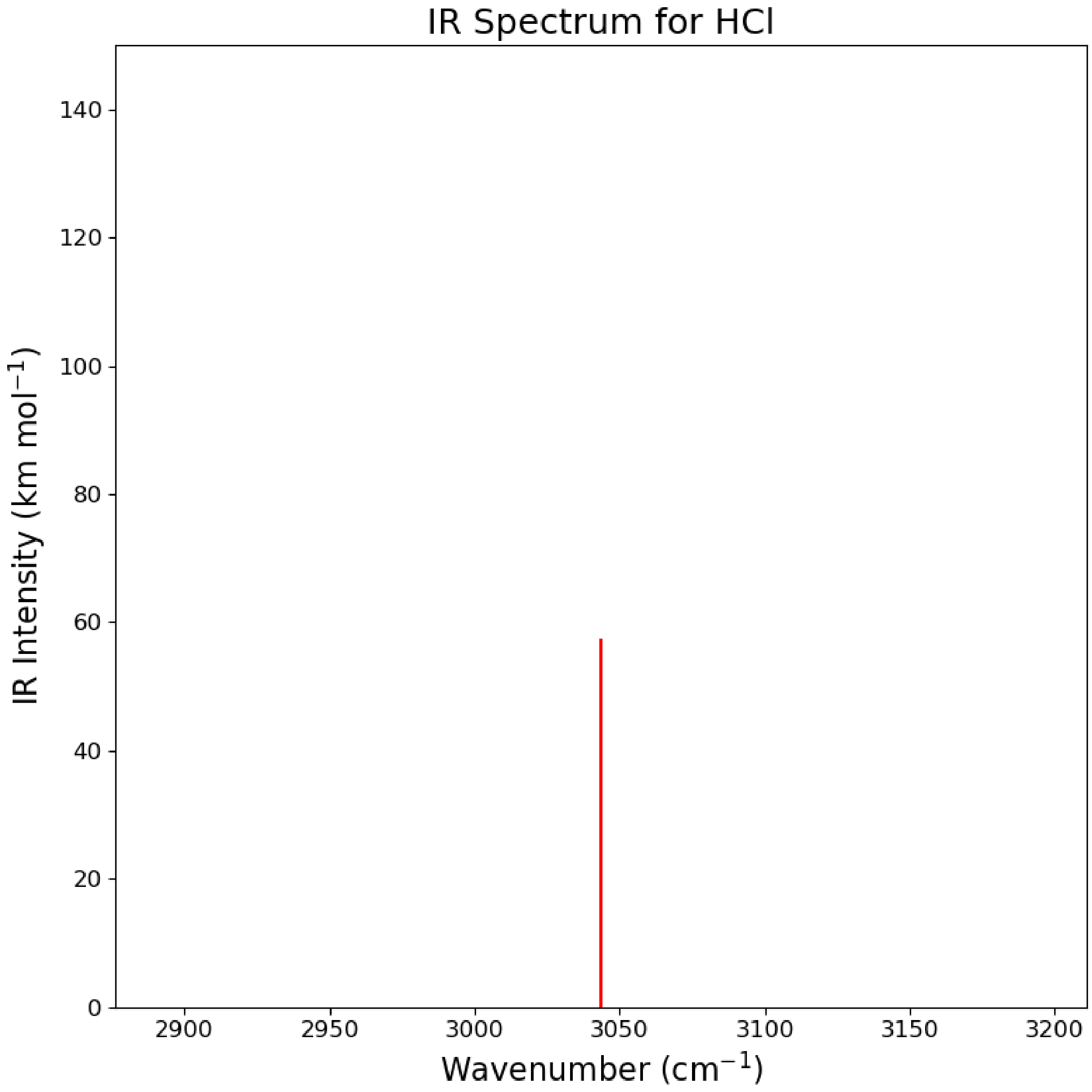

Fig S18. IR spectrum of HCl at the MP2-F12/aVDZ level of theory computed using AD.

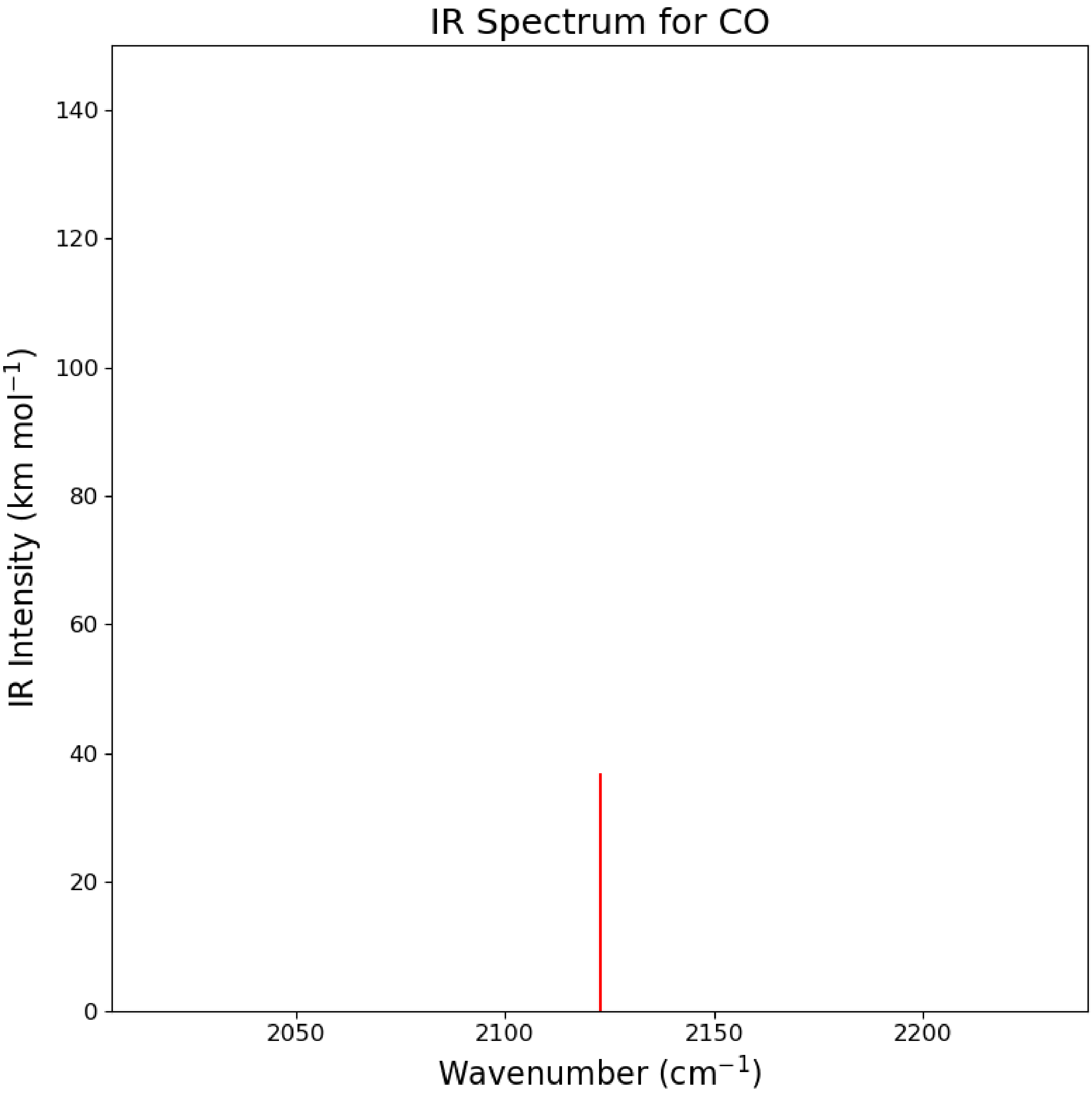

Fig S19. IR spectrum of CO at the MP2-F12/aVDZ level of theory computed using AD.

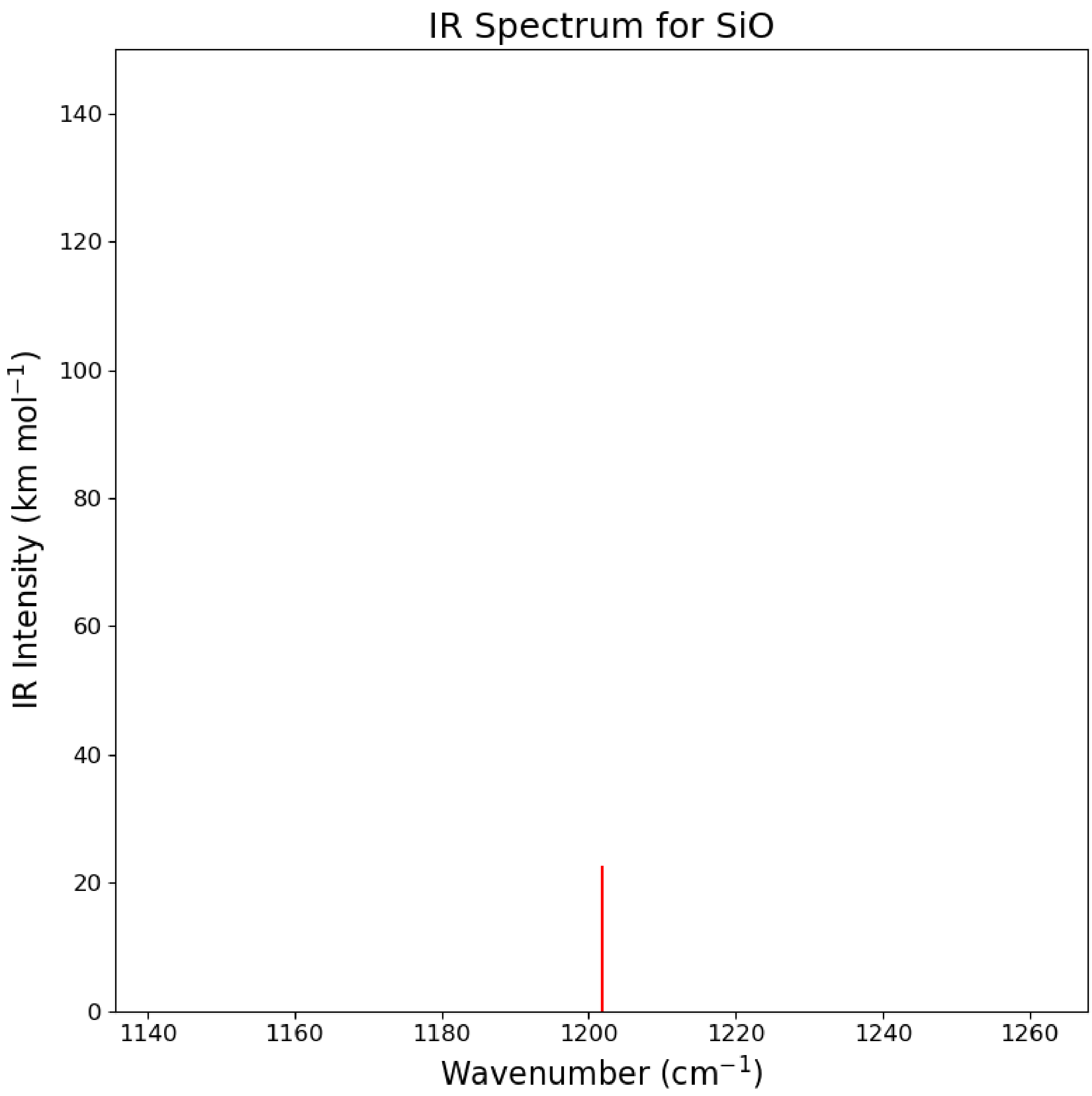

Fig S20. IR spectrum of SiO at the MP2-F12/aVDZ level of theory computed using AD.

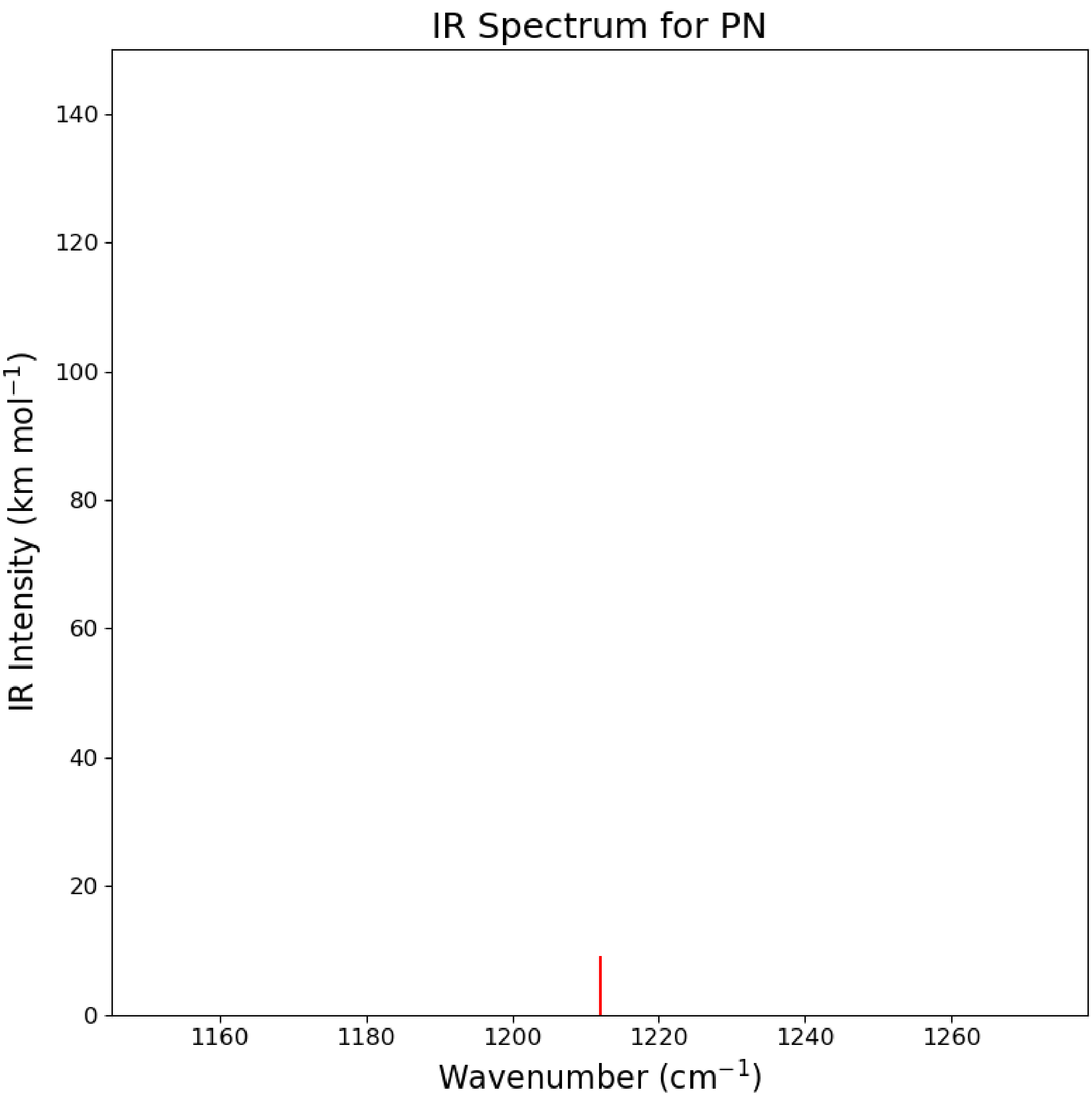

Fig S21. IR spectrum of PN at the MP2-F12/aVDZ level of theory computed using AD.

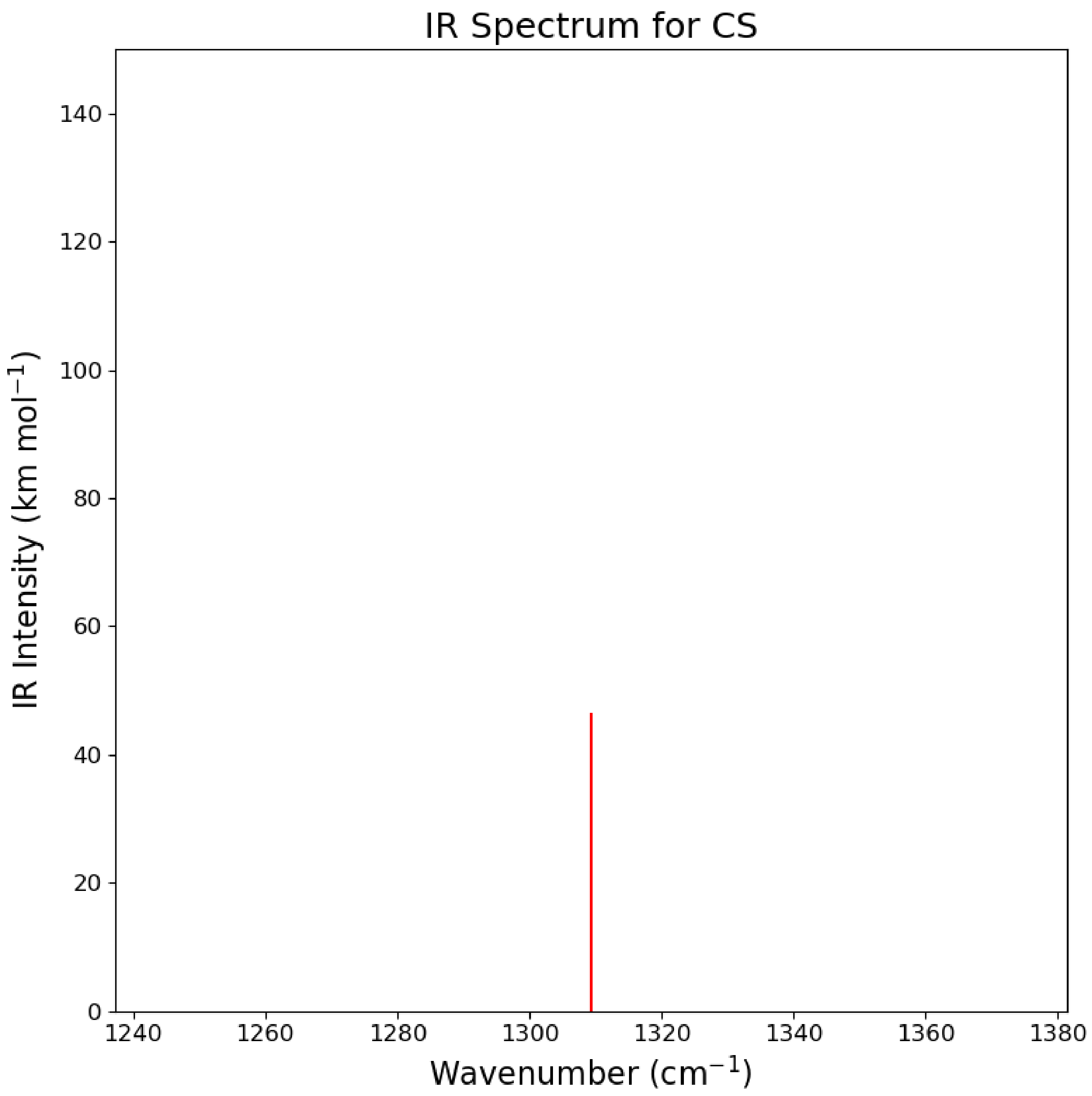

Fig S22. IR spectrum of CS at the MP2-F12/aVDZ level of theory computed using AD.
